# Cancer-Associated Adipocytes and Breast Cancer: Intertwining in the Tumor Microenvironment and Challenges for Cancer Therapy

**DOI:** 10.3390/cancers15030726

**Published:** 2023-01-24

**Authors:** Chenghui Wu, Shuwen Dong, Renhong Huang, Xiaosong Chen

**Affiliations:** Department of General Surgery, Comprehensive Breast Health Center, Ruijin Hospital, Shanghai Jiao Tong University School of Medicine, Shanghai 200025, China

**Keywords:** breast cancer, cancer-associated adipocytes, cytokines, adipokines, tumor microenvironment, treatment resistance

## Abstract

**Simple Summary:**

Breast cancer is the most prevalent cancer in women worldwide, and it exhibits a growing incidence. An increasing number of studies showed the complex bidirectional regulation between breast cancer and adjacent cancer-associated adipocytes. The present review summarizes the mechanisms of cancer-associated adipocyte formation in the breast cancer tumor microenvironment and the effect of cancer-associated adipocytes on the tumorigenesis, progression, and metastasis of breast cancer. We focused on the therapeutic resistance of breast cancer caused by cancer-associated adipocytes and potential strategies targeting cancer-associated adipocytes in breast cancer treatment.

**Abstract:**

Adipocytes are the main components in breast tissue, and cancer-associated adipocytes (CAAs) are one of the most important components in the tumor microenvironment of breast cancer (BC). Bidirectional regulation was found between CAAs and BC cells. BC facilitates the dedifferentiation of adjacent adipocytes to form CAAs with morphological and biological changes. CAAs increase the secretion of multiple cytokines and adipokines to promote the tumorigenesis, progression, and metastasis of BC by remodeling the extracellular matrix, changing aromatase expression, and metabolic reprogramming, and shaping the tumor immune microenvironment. CAAs are also associated with the therapeutic response of BC and provide potential targets in BC therapy. The present review provides a comprehensive description of the crosstalk between CAAs and BC and discusses the potential strategies to target CAAs to overcome BC treatment resistance.

## 1. Introduction

**Figure 1:**
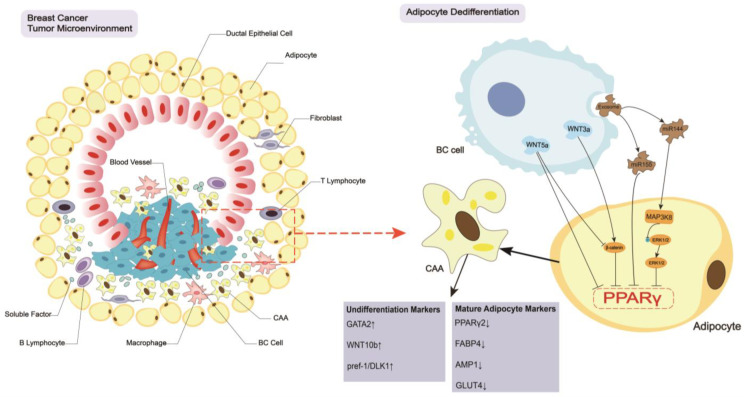
Dedifferentiation of adipocytes in the breast cancer TME. There are many components in the TME, including adipocytes, BC cells, CAAs, T lymphocytes, B lymphocytes, macrophages, small vessels, ductal epithelial cells, fibroblasts, and many soluble factors. Adipocytes adjacent to BC cells dedifferentiate and form CAAs via the inhibition of PPARγ by BC secretions, such as WNT proteins and exosomes. CAAs have an irregular shape and are filled with tiny distributed lipid droplets. Undifferentiation markers in CAAs (WNT10b, Pref-1/DLK1, and GATA2) increase, and mature adipocyte markers (PPARγ2, FABP4, APM1 and GLUT4) decrease. Abbreviations: BC, breast cancer; CAAs, cancer-associated adipocytes; TME, tumor microenvironment; miR144, microRNA 144; miR155, microRNA 155; PPARγ, peroxisome proliferator-activated receptor gamma; ERK1/2, extracellular-regulated kinase 1/2; MAP3K8, mitogen-activated protein kinase kinase kinase 8; GATA, GATA binding protein; WNT, wingless-related integration site; pref-1, preadipocyte factor 1; DLK1, delta-like 1 homologue; FABP4, fatty acid binding protein 4; AMP1, adenosine monophosphate 1; GLUT4, glucose transporter 4.

Breast cancer (BC) is the most prevalent cancer in women worldwide, and it has become the leading cause of cancer death in women. A recent epidemiological survey showed that BC surpassed lung cancer as the most common cancer worldwide in 2020 [1]. The five-year survival rate for BC has surpassed many other cancers due to early identification and the expanding availability of treatment targets and medications. However, considering the high prevalence, there is much research to be done. Recent research indicates that the tumor microenvironment (TME) and the BC cells themselves have a deep and extensive impact on the biological behavior and malignant development of the tumor [2] (Figure 1). A variety of cell types, including fibroblasts, immune cells, adipocytes, and vascular endothelial cells, and their abundant secretion of soluble factors, are present in the TME, which is highly complex, heterogeneous, and spatiotemporally variable. Additionally, since the content of intratumor adipocytes of BC is limited in the TME, peritumor adipocytes play a major role in this pathological process. Generally speaking, tumor cells reprogram the TME via paracrine and autocrine secretion, and the reprogrammed TME supports tumor development, invasion, angiogenesis, and metastasis [3,4,5,6]. Adipocytes have a significant impact on the TME of BC. To discover new diagnostic and therapeutic pathways, it is crucial to further comprehend and investigate the role of adipocytes in the TME in the development, growth, and metastasis of BC.

Adipocytes are a main element of the interstitial mammary gland. Breast adipocytes are divided into three groups: mature normal cells, preadipocytes, and adipose-derived stem cells (ADSCs) [7]. ADSCs have multilineage differentiation potential, which means that these cells can differentiate into several lineages, such as fat, bone, cartilage, skeletal, smooth, and cardiac muscle, endothelium, hematopoietic cells, hepatocytes, and neuronal cells. Preadipocytes, which belong to the fat lineage, undergo growth arrest, clonal expansion, and terminal differentiation into mature adipocytes [8,9]. Mature adipocytes that surround the mammary glands are crucial for preserving their healthy shape and energy supply [10]. Normal and abnormal physiological processes in the mammary gland (including tumorigenesis, progression, and metastasis) are linked to the surrounding adipocytes, and growing evidence shows that these adipocytes and their surroundings have complex bidirectional regulatory functions [11]. Many clinical studies showed that obesity was associated with higher incidence and poorer survival of BC, which sustains that adipocytes play a vital role in BC progression [12,13]. Therefore, adipocytes modified by tumor cells in the TME are referred to academically as cancer-associated adipocytes (CAAs) rather than normal adipocytes (Figure 1). CAAs may affect the biological behavior of tumor cells in a variety of ways. BC cells also regulate the formation of CAAs and influence the metabolism, secretion, and other physiological functions of CAAs. This mutual regulation is called as the “crosstalk” between BC cells and CAAs [7,11,14,15]. The current article specifically describes how this crosstalk affects the development, progression, metastasis, and therapeutic resistance of BC in the TME and developments in the treatment of BC linked to CAAs.

## 2. Breast Cancer Regulates the CAA Formation and Biology

### 2.1. Dedifferentiation of Adipocytes

Peroxisome proliferator-activated receptor gamma (PPARγ) is a member of the nuclear protein receptor superfamily. PPARγ and the co-activator CCAAT/enhancer binding protein α (C/EBPα) form a positive regulatory loop during adipocyte differentiation from mesenchymal stem cells [16]. These two transcription factors play important roles in regulating adipocyte differentiation, catabolism, and insulin sensitivity [17,18]. Adipocytes differentiate and dedifferentiate by controlling PPARγ and C/EBPα expression via various signaling pathways, which lead to different physiological and pathological expression. The so-called CAAs are also formed based on the dedifferentiation of adipocytes primarily via the following related mechanisms: WNT/β-catenin signaling pathway, WNT-PCP signaling pathway (non-canonic WNT pathway), and tumor-derived exosomes (Figure 1).

The most vital and well-studied regulated mechanism is the WNT/β-catenin signaling pathway. WNTs are a highly conserved family of autocrine and paracrine ligands, and different kinds of elevated WNT ligands are detected in different types of BC cells, such as WNT1, WNT2, WNT3, WNT3a, WNT5a, WNT5b, WNT6, WNT9a, WNT10a, and WNT10b [19]. WNT proteins bind to transmembrane receptors of the frizzled family and decrease β-catenin complex phosphorylation, and induce the stabilization and translocation of β-catenin to the nucleus. β-catenin interacts with T-cell factor/lymphoid enhancer factor (TCF/LEF) transcription factors to block PPARγ and C/EBPα expression, which inhibits 3T3-L1 and 3T3-F442A adipocyte differentiation [20]. Notably, WNT3a increases the levels of β-catenin to suppress the activation of PPARγ, which leads to adipocyte dedifferentiation and CAA formation. When 3T3-L1 adipocytes and human adipocytes were cultured in WNT3a-conditioned culture medium, several characteristic undifferentiated cell markers, including WNT10b, Pref-1/DLK1, and GATA2, were increased, and typical mature adipocyte markers decreased, such as PPARγ2, FABP4, APM1 and GLUT4 [21]. In addition to the classical WNT/β-catenin signaling pathway, the WNT-PCP pathway exists as the non-canonic WNT pathway. For example, WNT5A is elevated in basal-like BC, which inhibits the activation of PPARγ in 3T3-L1 adipocytes and promotes the dedifferentiation of adipocytes. WNT5A-neutralizing antibodies and a recombinant competitive WNT5A receptor inhibitor that secretes frizzled-related protein (SFRP5) reverses the dedifferentiation of adipocytes and promotes adipogenesis [19,22]. However, WNT5a antagonizes WNT/β-catenin signaling by promoting GSK-3-independent degradation of β-catenin [23], which antagonizes adipocyte dedifferentiation. Therefore, more experiments must be performed to make conclusions.

In addition to WNT-regulated signaling pathways in the formation of CAAs, exosomes play a vital role in CAA formation. Exosomes induce adipocyte dedifferentiation and CAA formation by regulating PPARγ in an autocrine or paracrine manner. For example, when the BC cells MCF-7 and MDA-MB-231 and the adipocytes 3T3-L1 were co-cultured, the highly secreted exosomal microRNA-144 (miR-144) increased the development of CAAs by binding with mitogen-activated protein kinase kinase kinase 8 (MAP3K8), which lowered extracellular-regulated kinase 1/2 (ERK1/2) phosphorylation and PPARγ levels to promote adipocyte dedifferentiation [24]. BC cell-derived exosomal miR-155 also downregulated PPARγ expression in adipocytes in another model, which led to adipocyte dedifferentiation [25]. The exosomes between adipocytes and BC cells are reciprocal. Exosomes released from ADSC-differentiated adipocytes increase BC cell proliferation and metastasis by activating the Hippo signaling pathway [26].

### 2.2. Biological Changes in CAAs

**Figure 2:**
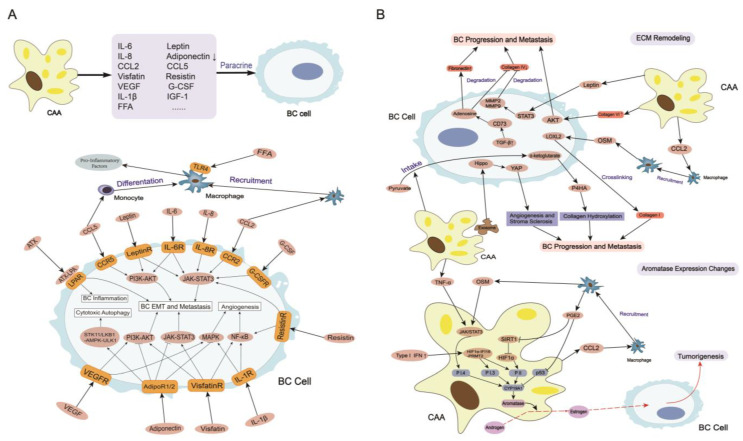
(**A**) CAAs secrete multiple cytokines and adipokines that modify the behavior of BC cells. CAAs secrete IL-6, IL-8, leptin, CCL2, CCL5, G-CSF, VEGF, IL-1β, ATX, resistin and visfatin to cause EMT, angiogenesis, and inflammation in BC by activating multiple signaling pathways, which promote BC progression and metastasis. CAAs secrete CCL2 and FFA to recruit and activate macrophages that release pro-inflammatory factors. CAAs reduce the secretion of adiponectin compared to normal adipocytes, which promotes tumor cell cytotoxic autophagy and BC metastasis. (**B**) CAAs remodel the BC extracellular matrix and change aromatase expression. CAAs regulate the content and physiological status of different collagens to remodel the ECM by increasing the secretion of leptin, collagen, OSM and some exosomes. Remodeled ECM is crucial to BC progression and metastasis. CAAs secrete CCL2, which recruits macrophages to release PGE2 and OSM. Increased type I IFN, TNF-α, PGE2, and OSM upregulate the expression of CYP19A1-encoded aromatase. Increased aromatase upregulates the expression of estrogen, which promotes the tumorigenesis of BC cells. Abbreviations: BC, breast cancer; CAAs, cancer-associated adipocytes; IL-6, interleukin-6; IL-8, interleukin-8; CCL2, C-C motif chemokine ligand 2; C-C motif chemokine receptor 2, CCR2; CCL5, C-C motif chemokine ligand 5; C-C motif chemokine receptor 5, CCR5; G-CSF, granulocyte colony-stimulating factor; VEGF, vascular endothelial growth factor; IL-1β, interleukin-1 beta; ATX, autotoxin; AdipoR1/2, adiponectin receptor1/2; TLR4, toll like receptor 4; FFA, free fatty acids; LAP, lysophosphatidic acid; PI3K, phosphatidylinositide 3-kinase; AKT, protein kinase B; STAT, signal transducer and activator of transcription; JAK, janus tyrosine kinase; EMT, epithelial mesenchymal transition; ECM, extracellular matrix; MAPK, mitogen-activated protein kinase; NF-κB, nuclear factor-kappa B; STK11, serine/threonine kinase 11; LKB1, liver kinase B1; AMPK, AMP-activated protein kinase; ULK1, Unc-51 like kinase 1; MMP, matrix metalloproteinase; YAP, Yes-associated protein; OSM, oncostatin M; P4HA, prolyl-4-hydroxylase; TNF-α, tumor necrosis factor alpha; PGE2, prostaglandin E2; IFN, interferon; SIRT1, silent mating type information regulation 2 homolog-1; HIF1α, hypoxia-inducible factor 1 alpha; IFI16, HIF1α–IFN-γ-inducible protein 16; PRMT2, protein arginine N-methyltransferase 2; TGF-β, transforming growth factor beta; LOXL2, lysyl oxidase-like 2; CD73, cluster differentiation 73; CYP19A1, cytochrome P450, family 19, subfamily A, polypeptide 1.

The decreased expression of PPARγ results in the dedifferentiation of adipocytes and the formation of CAAs. These cells have an irregular shape and are filled with tiny distributed lipid droplets [27] (Figure 1). CAAs reorganize the actin cytoskeleton and increase fibroblast-like biomarkers, such as fibroblast activation protein A, chondroitin sulfate proteoglycan, and smooth muscle actin, to develop a fibroblast-like morphology [28], which contributes to tumor cell metastasis. CAAs accelerate several metabolic processes by releasing metabolites, such as free fatty acids (FFAs), lactate, ketone bodies, pyruvate, glutamine and arginine, to meet the metabolic demands of tumor cells [29]. CAAs increase the expression of aromatase to upregulate the levels of estrogen in the breast cancer TME, which promotes the growth and progression of ER+ BC [30]. CAAs exhibit a malignant phenotype that is characterized by the increased secretion of leptin, interleukin-6 (IL-6), C-C motif chemokine ligand 2 (CCL2), C-C motif chemokine ligand 5 (CCL5), interleukin-beta (IL-1β), autotaxin (ATX), interleukin-8 (IL-8), vascular endothelial growth factor (VEGF), granulocyte colony-stimulating factor (G-CSF), visfatin and resistin (Figure 2A), which facilitates BC tumor development, progression, and metastasis.

## 3. CAAs Induce Breast Cancer Tumorigenesis, Progression, and Metastasis

### 3.1. Secretome in CAAs

#### 3.1.1. Leptin

Leptin is a key adipokine that is primarily produced by mature adipocytes, and it is enriched in breast adipocytes. Leptin production and secretion are substantially elevated in CAAs compared with mature adipocytes, and leptin receptors are highly expressed in BC epithelial cells compared with non-cancerous breast epithelial cells [31]. Leptin and leptin receptors regulate multiple processes associated with BC tumorigenesis, progression, angiogenesis, epithelial mesenchymal transition (EMT), metastasis, and immunosuppression. Elevated serum levels of leptin are positively associated with the risk, invasiveness, and poor prognosis of BC [31,32]. The following mechanisms are possible explanations for this association: Leptin activates janus tyrosine kinase (JAK)/signal transducer and activator of transcription 3 (STAT3) and cAMP response element (CRE) signaling pathways to upregulate cyclin D1, and downregulates cyclin-dependent kinase inhibitor p21 expression, which decrease cells in G0/G1 phase and increase cells in S phase in BC to promote BC cell proliferation and regulate apoptosis [33]. In vitro experiments showed that leptin acted on phosphatidylinositide 3-kinases (PI3K)/protein kinase B (AKT) signaling pathway to promote the mesenchymalization of BC cell lines, which promoted BC proliferation and metastasis [34]. Leptin significantly inhibits the immune killing effects of cluster of differentiation 8 positive (CD8+) T cells by activating the STAT3-fatty acid oxidation (FAO) axis and reducing glycolysis [35]. Leptin activates STAT3 via miR-34a-dependent and miR-34a-independent mechanisms to promote fibrinogen inhibitor pai-1-mediated BC metastasis [36]. Leptin also promotes the angiogenesis of BC cells by increasing the expression of vascular endothelial growth factor (VEGF) and vascular endothelial growth factor receptor type 2 (VEGFR2) in mouse mammary cells [37]. The interaction between leptin and estrogen expression is also an important factor in promoting BC growth. For example, leptin activates cAMP response element binding (CREB) protein-dependent aromatase expression by upregulating cyclooxygenase-2 (COX-2) expression [38].

Clinically, circulating estradiol and leptin play important roles in the risk of postmenopausal BC [39]. Another clinical trial suggested that a modest amount of weight loss reduced leptin levels, which may be associated with a lower BC risk in postmenopausal women [40]. Moreover, elevated levels of serum leptin were associated with an elevated risk of invasive BC in postmenopausal women with normal BMI [41]. A randomized control trial found that exercise decreased the fat tissue-to-total tissue mass premenopausal women, which decreased serum levels of leptin and BC risk [42]. These results show that leptin is associated with a high risk of BC in premenopausal and postmenopausal women.

#### 3.1.2. Adiponectin

Adiponectin is one of the most extensively studied adipokines, and it is primarily secreted by mature adipocytes. Adiponectin secretion is lower in CAAs than normal adipocytes [43]. Adiponectin has numerous effects on many different target tissues, such as an anti-apoptotic, anti-inflammatory, anti-fibrotic and insulin sensitizing agent effect [27]. Female BC tumors with low circulating adiponectin levels have more aggressive features, such as higher histological grade and an enhanced tendency of angiogenesis and metastasis [44]. Adiponectin also has anti-proliferative and anti-metastatic functions in BC, which are exerted via the following relevant mechanisms: adiponectin binds to adiponectin receptors AdipoR1 and AdipoR2 to inhibit BC cell growth and migration by activating AMP-activated protein kinase (AMPK) and inhibiting the PI3K/AKT/mammalian target of rapamycin (mTOR) and c-Src (proto-oncogene)/mitogen-activated protein kinase (MAPK) signaling pathways [45]. Adiponectin inhibits leptin signaling by downregulating STAT3 activation, AKT phosphorylation, and WNT signaling by upregulating the suppressor of cytokine signaling 2, which inhibits BC cell proliferation and metastasis [45]. Moreover, adiponectin inhibits leptin signaling by downregulating sterol regulatory element-binding protein 1 (SREBP-1) and FAS (CD95)-related enzymes to inhibit fatty acid synthesis in BC cells. Inhibition of fatty acid synthesis stimulates adipocyte autophagy-mediated lipolysis and FAO to induce BC cell death [46]. Adiponectin leads to cytotoxic autophagy in BC cells by activating the STK11/liver kinase B1 (LKB1)-mediated AMPK-Unc-51 like kinase 1 (ULK1) axis [47]. A systematic meta-analysis showed that low circulating adiponectin levels were associated with an increased BC risk in postmenopausal and premenopausal women [48]. High levels of adiponectin were associated with a decreased risk of BC, which indicated that adiponectin may play a vital role in the etiology of BC [49].

#### 3.1.3. IL-6

IL-6 is a complex and multifunctional cytokine involved in pathophysiological processes, such as inflammation, immune response, hematopoiesis, and tumorigenesis [50]. The interaction between CAAs and BC cells results in increased IL-6 secretion from CAAs, and excess IL-6 leads to BC cell proliferation, EMT, and metastasis via binding to the receptor IL-6R [51]. IL-6 and leptin secreted by adipocytes activate the JAK/STAT3 and PI3K/AKT signaling pathways to enhance lysyl hydroxylase (PLOD2) expression, which promotes BC metastasis in vivo [52]. IL-6 promotes monocyte THP-1 polarization into M2-type macrophages in the TME in triple negative BC (TNBC), which promotes the BC progression [53]. The IL6/STAT3 signaling pathway enhances the expression of SNAIL by hijacking ER enhancers to drive a unique transcriptional program that promotes BC metastasis in estrogen receptor-positive (ER+) BC [54]. Clinically, serum IL-6 concentrations were significantly higher in patients with breast cancer compared with healthy controls, and IL-6 serum levels were significantly higher in patients with metastatic breast cancer than patients without metastasis [55]. High levels of serum IL-6 had independent prognostic value in BC because circulating IL-6 levels were associated with worse survival in patients with metastatic BC [56]. Moreover, serum IL-6 levels were feasible in assessing the efficacy of chemotherapy because a consistent decline in serum IL-6 levels was observed in locally advanced BC patients with chemotherapy [57]. These clinical studies suggest that IL-6 is a diagnostic and therapeutic evaluation indicator of BC.

#### 3.1.4. CCL2

CCL2 is encoded by the CCL2 gene located on chromosome 17q12, and it binds to C-C motif chemokine receptor 2 A (CCR2A) and C-C motif chemokine receptor 2 B (CCR2B) to regulate a range of physiological and pathological processes [58]. Increased secretion of CCL2 in CAAs has been observed in mouse BC models and BC patients [59], and it promotes BC metastasis by promoting angiogenesis and EMT via activation of the STAT3 signaling pathway [60]. CCL2 recruits circulating macrophages in the TME to form a crown-like structure, which leads to the release of a series of inflammatory mediators that promote tumor angiogenesis and immunosuppression [59,61]. The oroxylin A inhibitor ACTNI reduces the secretion of CCL2 by inhibiting phosphorylation of the focal adhesion kinase (FAK)/STAT3 signaling pathway, which inhibits BC metastasis [62]. Clinically, high CCL2 expression was associated with a decreased overall survival rate in BC patients [60], and high endocrine CCL2 increased the aggressiveness of human inflammatory BC and led to a poor prognosis [63].

#### 3.1.5. CCL5

CCL5, originally known as RANTES, is encoded by the CCL5 gene located on chromosome 17q12. CCL5 is an important chemokine that is primarily secreted by tumor stromal cells under the stimulation of BC cells, and it is closely associated with the invasiveness and metastasis of BC [64,65]. When co-cultured with BC cells, CAAs increased the secretion of CCL5, which promoted the EMT and metastasis of BC [66]. In vitro experiments showed that CLL5 bound to the receptor CC5R on monocyte THP-1 cells, which differentiated into TAMs. These TAMs were recruited to the TME and generated a series of inflammatory responses that promoted the proliferation and metastasis of the MCF-7 and MDA-MB-231 BC cell lines [67]. CCL5 promotes the production of immunosuppressive myeloid cells and TAMs, and immunosuppressive myeloid cells reduce tumor-infiltrating cytotoxic CD8+ T cells and elevate regulatory T cells in the tumor-draining lymph nodes, which leaves the TME in an immunosuppressed state [68]. CCL5 mediates Th2 (IL4⁺ CD4⁺ T) cell polarization, which promotes metastasis in luminal BC [69]. When CCL5 combines with CCR5 on BC cells, the mTOR/AKT signaling pathway is activated, which increases the angiogenesis and metastasis of BC [70]. Clinically, CCL5 was associated with poor disease-free survival and overall survival in patients with early human epidermal growth factor receptor 2-positive (HER2+) BC. The abundance of CCL5 in peritumoral adipose tissue of TNBC patients was also associated with metastasis and poorer overall survival [71].

#### 3.1.6. IL-1β

IL-1β is a pro-inflammatory cytokine in BC that is secreted by monocytes, dendritic cells (DCs), macrophages, CAAs, and tumor cells [72]. When co-cultured with BC cells, CAAs increased the secretion of IL-1β, which is an important factor and driver of tumor malignance [73]. IL-1β plays a key role in promoting the different stages of bone metastasis in BC by affecting primary tumor growth and angiogenesis, promoting tumor cell movement into the circulating blood, and promoting tumor cell colonization in the bone microenvironment [72]. IL-1β leads to the production of CXCL9 and CXCL10 in lung fibroblasts via the nuclear factor-kppa B (NF-κB) signaling pathway, which promotes lung metastasis of BC. Therefore, IL-1β is a predictive biomarker for an increased risk of bone metastasis and lung metastasis in BC. Inhibition of IL-1β inhibits tumor growth and proliferation and reduces the risk of bone, lung, and other site metastasis of BC [72,74,75]. In obese patients, adipocyte-derived IL-1β activates NF-κB and MAPK to induce angiopoietin-like 4 (ANGPTL4) expression in BC cells, which increases angiogenesis and BC progression [76]. Therefore, ANGPTL4 is a potential therapeutic target for obese BC patients. Clinically, higher serum IL-1β levels were associated with higher BC risk in post-menopausal patients [77]. Moreover, IL-1β was related to tumor cell release into circulation in BC patients, which was associated with reduced disease-free survival, breast cancer-specific survival, and overall survival [78].

#### 3.1.7. Other Adipokines and Cytokines

In addition to the adipokines mentioned above, the interaction between CAAs and BC cells involves a variety of other adipokines, such as resistin, visfatin, ATX and SFRP5. Visfatin and resistin are two adipocyte-derived factors that are elevated in BC patients, especially obese BC patients, and play important roles in promoting BC proliferation and metastasis. Visfatin induces a G1-to-S cell cycle transition by activating tumor survival signaling pathways such as NF-κB/Notch1, c-Abl/STAT3 and AKT/ERK1/2. Resistin promotes BC proliferation and metastasis by upregulating the expression of BCL-2 and BCL-XL and activating STAT3 and Toll-like receptor 4 (TLR4)-mediated NF-κB signaling pathways [79]. Previously, we found that resistin was a functional downstream target of TAZ (a transcription cofactor) that facilitated tumorigenesis of BC by increasing tumor stemness [80]. Adipocytes and ADSCs are the main producers of ATX, which is the secretory state of lysophosphatidic acid (LPA)-producing phospholipase D. The ATX-LPA signaling pathway plays an important role in promoting BC inflammation and enhancing BC aggressiveness [81]. The expression of SFRP5 increases during the differentiation and maturation of adipocytes, and CAAs reduce the secretion of SFRP5, which promotes BC cell migration and invasion via the WNT and EMT signaling pathways [82,83].

Many other cytokines secreted by CAAs play vital roles in BC development, such as IL-8, HGF, G-CSF, VEGF, insulin-like growth factor 1 (IGF-1), and leukemia-inhibitory factor (LIF). IL-8 is a major paracrine mediator of pro-carcinogenesis in breast adipocytes, and IL-8 released from CAAs in BC activates the STAT3 axis, which increases the angiogenesis of BC and enhance metastatic capacity [84]. Moreover, IL-8 recruits neutrophils into the breast cancer TME and the secretion of multiple cytokines to promote angiogenesis and inflammation in BC [85]. Hepatocyte growth factor (HGF) is a potent mitogenic and angiogenic factor that is secreted by CAAs [86]. HGF activates the c-Src/MAPK and STAT3 signaling pathways, which promote early BC formation [87]. Clinically, higher levels of HGF were significantly associated with shorter relapse-free and overall survival in BC patients compared with patients with lower levels of HGF [88]. CAA-derived granulocyte-colony stimulating factor (G-CSF) promotes BC cell migration and invasion by activating the STAT3 signaling pathway [89]. G-CSF regulates arginase 1 in myeloid cells to promote an immunosuppressive TME, which promotes BC metastasis [90]. VEGF is a vital angiogenic factor that is secreted by CAAs. When binding with VEGF receptors, VEGF promotes the proliferation, migration, and angiogenesis of BC via activating the MAPK and PI3K/AKT signaling pathways [91]. Clinically, a higher level of VEGF was associated with a short progression-free survival and postrelapse overall survival in BC patients [92]. Adipocytes increased the secretion of IGF-1 when co-cultured with BC cells, which increased the expression of fatty acid synthase (FASN) in tumor cells and promoted the growth and metastasis of BC [93,94]. CAA-derived LIF promotes the migration and invasion of BC cells via the STAT3 signaling pathway. The activation of STAT3 also promotes the secretion of C-X-C subfamily chemokines (CXCLs) in BC cells, which activates the ERK1/2, NF-κB and STAT3 signaling cascade to promote the expression of LIF in CAAs [95].

### 3.2. ECM Remodeling

The extracellular matrix is composed of fibrillar collagens, fibronectin, specific laminins, proteoglycans and matricellular proteins. The content and proportion of different collagens control the compliance, stiffness, porosity, viscoelasticity, and biochemical properties of the matrix in cancer. The collagen-rich and laminin-rich basement membrane and basement membrane stroma constitute the boundary of tumor cells, and tumor cells metastasize only if they penetrate through the basement membrane [96], which is regulated by CAAs (Figure 2B). CAAs change the content of multiple components in the ECM, such as collagen I, collagen IV, collagen VI, and fibronectin, by secreting many adipokines and cytokines, which influence the progression and metastasis of BC. Specifically, TAMs recruited by CAAs secrete oncostatin M (OSM) to upregulate lysyl oxidase-like 2, which increases the cross-linking of collagen I fibers in the ECM, and promotes IDC invasion and metastasis [85,97]. Leptin produced by adipocytes promotes the secretion of MMP-2 and MMP-9 by activating STAT3 signaling pathways, which degrade collagen IV, promote basement membrane rupture, and facilitate metastasis of BC [98]. CAAs secrete and process soluble ECM protein collagen VI, which activates NG2/chondroitin sulfate proteoglycan receptors on the surface of malignant ductal epithelial cells, activates AKT, β-catenin and cyclin 1 and promotes the EMT and metastasis of BC [99]. When co-cultured with adipocytes, elevated transforming growth factor-β (TGF-β) in BC promoted CD73 expression, which increased the adenosine content and resulted in a decrease in collagen IV and an increase in fibronectin in the ECM to ultimately promote tumor growth and metastasis of BC [100]. A recent study found that exosomes secreted by CAAs activated Hippo signaling pathways, which promoted stroma sclerosis and induced angiogenesis in the ECM to promote BC proliferation and metastasis [26]. CAAs reshape the metabolic niche of the ECM by altering the metabolism of BC cells to increase their uptake of pyruvate and the content of the metabolite α-ketoglutarate, which increase the activity of prolyl-4-hydroxylase and promotes the hydroxylation of collagen to ultimately promote BC metastasis [101]. Overall, CAAs play an important role in ECM remodeling primarily by regulating the content and physiological status of different collagen and fibronectin molecules, which would lead to BC progression and metastasis.

### 3.3. Aromatase Expression Changes

Estrogen is a sex steroid hormone that has important effects on tumorigenesis and regulation of the TME in BC. Estrogen acts directly and indirectly on DNA via metabolites that lead to DNA damage and impair DNA repair in BC. Estrogen also binds with the estrogen receptors (ERs) in ER+ breast cancers (more than 70% of breast cancers) to inhibit tumor cell autophagy and deregulate the cell cycle which lead to BC tumorigenesis [102]. Estrogen is primarily produced in white adipocytes in postmenopausal women because these cells convert androgen to estrogen via intrinsic aromatase. Furthermore, the levels of estrogen are higher in breast tissue than circulating estrogen in postmenopausal BC patients [103], and the activity of aromatase is significantly higher in the adipose tissue surrounding breast tumors than in other parts of the breast [104]. The expression of aromatase in adipocytes is primarily encoded by the human gene CYP19A1. Tissue-specific promoters PI.4, PII, and PI.3 regulate the expression of CYP19A1-encoded aromatase by regulating the relevant mRNA [105]. CAAs increase aromatase activity by releasing cytokines and adipokines, which primarily react on PI.4, PII, and PI.3 (Figure 2B). CAAs increase TNF-α and OSM levels, which stimulate PI.4 activity via the JAK1/STAT3 pathway and ultimately increase aromatase expression in the TME [85,105,106]. Chemokines released from adipocytes such as CCL2 recruit macrophages to increase the synthesis of prostaglandin E2 (PGE2) by secreting cyclooxygenase-2 (COX-2) and producing a chronic inflammatory response in the TME, which further increases aromatase expression by suppressing p53 (one kind of aromatase expression down-regulator) expression. PGE2 also down-regulates SIRT1 (a regulator of the cellular stress response and genomic integrity) to up-regulate and stabilize hypoxia-inducible factor 1 alpha (HIF-1α) levels, which leads to the binding and stimulation of PII and ultimately an increase in aromatase expression [105]. Type I IFN was elevated when adipocytes were co-cultured with BC cells which led to recruitment of the HIF-1α–IFN-γ-inducible protein 16 (IFI16)–protein arginine N-methyltransferase 2 (PMT2) complex to activate the PI.3/PII promoter in adipocytes and upregulate the expression of aromatase to promote the growth of ER+ BC [107,108]. Therefore, the Type I IFN-IFI16 axis may be an underlying therapeutic target. Briefly, adipokines and cytokines released by CAAs directly enhance aromatase expression or create an inflammatory TME to enhance aromatase expression which increases the levels of estrogen in the breast cancer TME promotes the growth of ER+ BC cells.

### 3.4. Metabolism Remodeling

**Figure 3:**
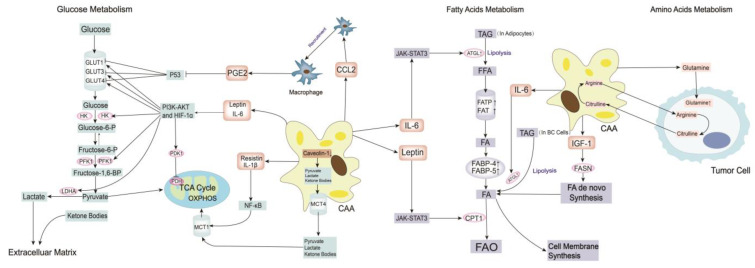
CAAs remodel BC metabolism. CAAs promote the expression of GLUT and the glycolysis rate-limiting enzymes HK and PFK1 to transport more glucose into BC cells and increase glycolysis in BC cells. CAAs promote the expression of LDH and PDK1 to increase lactate production and inhibit the TCA cycle in BC cells. CAAs promote the expression of MCT1 in BC cells to increase the uptake of pyruvate, lactate and ketone bodies, which are material for OXPHOS. Upregulated MCT4 in CAAs facilitates the efflux of pyruvate, lactate, and ketone bodies that are transported into adjacent BC cells as material of OXPHOS. CAAs promote the expression of ATGL in adipocytes and BC cells to increase lipolysis and produce more FFAs, which are energy resources and substrates for cell membrane synthesis. CAAs promote the expression of FAT, FATP, FABP4 and FABP5 to transport FFAs into BC cells. CAAs promote the expression of CPT1 to increase FAO to provide more energy. CAAs provide more glutamine for adjacent BC cells. CAAs take up citrulline in the extracellular matrix and convert it to arginine, which is consumed and decomposed to produce citrulline in tumor cells again. Abbreviations: BC, breast cancer; CAAs, cancer-associated adipocytes; IL-6, interleukin-6; IL-1β, interleukin-1 beta; TME, tumor environment; GLUT, glucose transporter; glucose-6-P, glucose-6-phosphate; fructose-6-P, fructose-6-phospahte; fructose-1,6-BP, fructose-1,6-biphosphate; HIF1α, hypoxia-inducible factor 1 alpha; HK, hexokinase; PFK, phosphofructokinase; LDHA, lactose dehydrogenase A; PI3K, phosphatidylinositide 3-kinase; AKT, protein kinase B; PDH, pyruvate dehydrogenase; PDK, pyruvate dehydrogenase kinase; MCT, monocarboxylate transport protein; TNF-α, tumor necrosis factor alpha; PGE2, prostaglandin E2; NF-κB, nuclear factor-kappa B; TCA, tricarboxylic acid cycle; OXPHOS, oxidative phosphorylation; LIF, leukemia inhibitory factor; TAG, triacylglycerol; ATGL, adipose triglyceride lipase; FFA, free fatty acids; FATP, fatty acid transporter protein; FAT, fatty acid translocase; FABP, fatty acids binding protein; CPT1, carnitine palmitoyl transferase 1; FAO, fatty acid oxidation; FASN, fatty acid synthase; JAK, janus tyrosine kinase; STAT, signal transducer and activator of transcription.

Extensive metabolic profile differences have been found between BC tissues and normal breast tissues. Adipocytes in breast tissue influence BC cells metabolism by secreting cytokines and adipokines, primarily glucose metabolism, fatty acid metabolism, and amino acid metabolism (Figure 3).

#### 3.4.1. Glucose Metabolism

Tumor cells prefer glycolysis under both aerobic and anaerobic conditions (“aerobic glycolysis” or “Warburg effect”), which quickly obtain ATP and other materials for macromolecular synthesis, such as acetyl-CoA and NADPH [109]. Glycolysis involves several key transporters and enzymes, including glucose transporter (GLUT), hexokinase (HK), phosphofructokinase (PFK), pyruvate dehydrogenase kinase (PDK), lactose dehydrogenase (LDH) and monocarboxylate transport proteins (MCTs), which are influenced by CAAs in the TME [24]. By increasing the secretion of leptin and IL-6, CAAs activate the PI3K-AKT axis and HIF-1α to increase GLUT1, GLUT3, GLUT4, HK1, HK2, PDK1, PFK1 and LDHA expression, which leads to metabolic reprogramming in BC cells, including upregulating the rate of glycolysis and increasing lactate production [110]. Adipocyte-derived CCL2 recruits macrophages into the breast cancer TME to form TAMs, which increase the secretion of PGE2. PGE2 inhibits the expression of the tumor suppressor p53, which increases the expression of GLUT1, GLUT3 and GLUT4, increases aerobic glycolysis and promotes the “Warburg effect” of BC. [111,112]. MCT4 is highly expressed in CAAs, and it transports lactate and ketone bodies out of the CAAs, which are then transported by MCT1 into the tumor cells to support OXPHOS (“reverse Warburg effect”) [113]. Adipocytes-derived resistin and IL-1β activate the NF-κB signaling pathway to promote MCT1 expression in BC cells, which facilitates the uptake of pyruvate, lactate and ketone bodies as energy resources and promotes the “reverse Warburg effect” [114]. Hypoxia in the TME leads to the deletion of caveolin-1 expression on peritumor mesenchymal cells and the promotion of aerobic glycolysis in adipocytes, which produce more lactate and pyruvate [115]. That act as worse prognostic markers of BC [116]. Uncoupling protein 1 (UCP1) is elevated in adipocytes adjacent to BC tumor cells, and its expression is found to be positively correlated with MCT4 expression. UCP1 plays a pivotal role in maintaining energy balance and increasing lipid mobilization in CAAs [114,117]. However, its effect on BC is controversial, and more research is needed. In general, CAAs increase the release of specific cytokines to activate the PI3K-AKT axis and HIF-1α or recruit TAMs to inhibit the expression of p53, which increase the expression of glucose transporters and glycolysis-related enzymes to promote the “Warburg effect” in BC cells. CAAs upregulate the expression of MCT1 on tumor cells and MCT4 on adipocytes to facilitate the “Warburg effect” and “reverse Warburg effect” of BC cells.

#### 3.4.2. Fatty Acid Metabolism

Fatty acids are enriched in adipocytes, and there is a complex metabolic symbiosis between adipocytes and BC cells. As mentioned above, tumor cells regulate CAA formation and make them morphologically irregular and filled with many small lipid droplets. Lipids are stored as intracellular triacylglycerol (TAG) in CAAs and are released from CAAs as free fatty acids, which need to undergo lipolysis catalyzed by different lipases, such as adipose triglyceride lipase (ATGL), hormone-sensitive lipase (HSL), and monoacylglycerol lipase (MAGL) [118]. CAAs increase the secretion of IL-6 to activate the JAK/STAT3 signaling pathway, which promotes ATGL-dependent lipolysis in adipocytes [119]. Fatty acids (FAs) are efficiently transported from the TME to tumor cells by surface transporters in BC cells, such as CD36, fatty acid translocase (FAT) and fatty acid transporter protein (FATP) [120]. Then BC cells transport FFAs via intracellular fatty acid transporters, such as fatty acid binding protein-4 (FABP-4) and FABP-5 [121,122]. The co-culture of adipocytes and MCF-7 and MDA-MB231 cell lines increased the expression of FAT, FATP, FABP-4 and FABP-5 in MCF-7 and MDA-MB231 cell lines, which transported more FAs into BC cells to provide energy. Overexpression of FAT in BC cells also induces stemness in BC by activating the STAT3 signaling axis, which inhibits BC cell autophagy and enhances BC cell invasiveness [123]. Once being transported into tumor cells, many FAs are stored as triglycerides in lipid droplets and undergo lipolysis to form FAs again under the catalysis of ATGL, HSL and MAGL [119]. CAAs increase the secretion of IL-6, which increases ATGL expression in BC cells and facilitates lipolysis in tumor cells to produce FAs as energy resources and material for macromolecule synthesis, which promotes BC aggressiveness [119,124]. FAs in BC cells undergo β-oxidation in the cytoplasm and mitochondria to produce NADH, flavine adenine dinucleotide (FADH2), and acetyl-COA, referred to as FAO, which is directly regulated by the rate-limiting enzyme carnitine palmitoyl transferase 1 (CPT1). Adipocyte-derived leptin promotes CPT1 expression by activating the JAK/STAT3 axis, which activates FAO and increases the aggressiveness and drug resistance of BC [35]. FASN is a key enzyme for FA de novo synthesis from glucose in tumor cells, and FAs are the basic material for the synthesis of phospholipids, which are the main component of the cell membrane in BC cells. When co-cultured with MCF-7 BC cell lines, mouse 3T3-L1 adipocytes and human mammary adipocytes increased the release of IGF-1, which increased the expression of FASN in tumor cells and promoted the growth and metastasis of BC [93,94]. FASN inhibitor G28UCM inhibited the growth of HER2+ BC and even in patients with acquired resistance to anti-HER2 drugs, which may provide a new way to treat trastuzumab-resistant BC [125]. Overall, CAAs promote the expression of ATGL both in adipocytes and BC cells, which accelerates lipolysis in these cells and produces more FAs as energy resources and material for cellular structure synthesis, such as cytomembranes. CAAs promote the expression of fatty acid transporters in BC cells to take up more FAs, and CAAs increase the rate of FAO in BC cells by promoting the expression of CPT1. CAAs increase the expression of FASN in tumor cells to facilitate FA de novo synthesis from glucose.

#### 3.4.3. Amino Acid Metabolism

Amino acids such as glutamine and arginine are important substrates for tumor cell growth and proliferation because they contribute greatly to both anabolism and catabolism, and act as signaling molecules in BC cells [126]. There is a tight symbiotic metabolic relationship between CAAs and BC cells in amino acid metabolism. One of the most important amino acids in BC cell metabolism is glutamine. CAAs synthesize glutamine and provide it for adjacent tumor cells, which promote the growth and metastasis of BC [127]. Adipocytes downregulate the expression of p62 and inhibit the mTORC1 signaling pathway to reduce adipocyte consumption of glutamine, which provides more glutamine for tumor cells [128]. Moreover, glutamine oxidation increased in BC cells when co-cultured with adipocytes, which promoted the growth progression of BC because CAAs provided much glutamine for BC cells, and the tumor cells had more glutamine as a substrate for catabolism [129]. Apart from glutamine, tumor cells consume and decompose arginine to produce citrulline, which is secreted into the extracellular matrix and taken up by CAAs in the matrix. Citrulline is converted back into arginine and used by tumor cells to promote the growth of ovarian and endometrial cancers. We hope there will be more research on this process in BC [130]. In general, the consumption of some typical amino acids such as glutamine increases substantially in BC. CAAs help the production and increase the utilization of these amino acids, which ultimately facilitate the growth and metastasis of BC.

### 3.5. Immune System Changes

The tumor immune microenvironment (TIME) is a relatively separate part of the tumor that constitutes a complex ecosystem with low antitumor immune activity, which is responsible for tumor growth and metastasis. Immune cells in the TIME include myeloid-derived suppressor cells (MDSCs, pathologically activated neutrophils and monocytes with potent immunosuppressive activity), TAMs, natural killer cells (NKs), lymphocytes, and DCs [131]. By interacting with the ECM, immune cells, BC cells and surrounding mesenchymal cells co-regulate the TIME. CAAs play a vital role in this process by increasing the release of inflammatory cytokines and adipokines, such as IL-8, IL-6, leptin, CCL2, CCL5, FAs, IL-1β, and VEGF [84]. More specifically, CAAs recruit and regulate different immune cells in the TIME, which promotes the progression and metastasis of BC.

#### 3.5.1. Tumor-Associated Macrophages

Macrophages are always enriched in the TIME of BC, called as tumor-associated macrophages (TAMs), which account for more than 50% of the tumor mass. TAMs are divided into two main categories, resident macrophages and recruited macrophages. Resident macrophages are derived from hematopoietic progenitors present in the capsule, and recruited macrophages arrive in the breast cancer TIME following the blood flow [132]. TAMs in the TIME of BC are polarized into two types, M1 and M2, under stimulation by different signals. M1 cells restrain tumor development, and M2 cells promote tumor development [133]. TAMs generally accumulate around dead adipocytes in the TIME and engulf them to form a crown-like structure, which is a histological hallmark of the pro-inflammatory process. These TAMs secrete multiple cytokines, such as IL-6, OSM, CSF-1, MMPs, VEGF, HIF-1α, and TGF-β which promote tumorigenesis, EMT, angiogenesis, metastasis, and drug resistance in BC cells [134]. Cytokines produced by TAMs, such as CSF-1, form a positive loop by facilitating the recruitment of new macrophages into the TIME and promoting macrophage polarization into the M2 phenotype [135]. CAAs play an important role in the recruitment and polarization of TAMs in the TIME. Firstly, CAAs increase the secretion of CCL2, which recruits circulating macrophages to BC tissue from the blood. Cellular free DNA released from degenerating adipocytes also increases the levels of CCL2 to promote the recruitment of TAMs [61,136]. Secondly, activation of the CLL5-CLL5R axis promotes the production of MDSCs and the differentiation of monocytes into TAMs [68]. Thirdly, CAAs increase lipolysis to release more FAs, which promote macrophage M2 polarization. By activating the ERK/STAT3 pathway, CAA-derived adenosine and lactate enhance M2 polarization [84,137]. Moreover, FAs in the breast cancer TIME are recognized by TLR4 on the cell surface of macrophages, which activates the NF-κB signaling pathway and increases the release of pro-inflammatory factors, such as PGE2 and OSM to subsequently cause local inflammation in the TME of BC [138]. This inflammation increases aromatase expression and promotes the growth and metastasis of BC [138]. Overall, CAAs recruit macrophages into the breast cancer TIME and promote macrophage polarization to an immunosuppressive phenotype, and CAAs increase pro-inflammatory factor secretion in TAMs, which leads to an inflammatory and immunosuppressive TIME in BC.

#### 3.5.2. Other Immune Cells

CAAs increase the secretion of IL-8 to recruit neutrophils to the TME in BC, and secrete cytokines, such as OSM and VEGF, to promote angiogenesis and inflammation in BC, which promote metastasis [85,139]. CAAs increase the secretion of IL-6 and leptin, which activate the JAK/STAT3 axis in BC cells and reduce the sensitivity of tumor cells to the toxic effects of NKs [84]. Released FAs also shut down the antitumor immune response and decrease the antitumor activity of NKs. FAs reduce mTORC1/c-MY signaling in NKs and the rates of glycolysis and OXPHOS, which results in the low production of interferon gamma (IFN-γ), granzyme B and perforin and reduced cytotoxicity to targeted tumor cells [140]. CAA-derived metabolites, such as FAs, adenosine and lactate, weaken the capability of DCs to process tumor antigens and effectively activate T cells [84]. As mentioned previously, leptin significantly inhibits the antitumor effects of CD8+ T cells by activating the STAT3/FAO axis and reducing glycolysis [35]. Programmed death-ligand 1 (PD-L1) expression in CAAs prevents anti-PD-L1 antibodies from activating important antitumor functions of CD8+ T cells, which contribute to the immune escape of BC cells [141]. These studies show that CAAs recruit and regulate immune cells, such as neutrophils, NKs, DCs, and T lymphocytes, to decrease the anti-tumor effect of the TIME, which ultimately promotes BC metastasis and cancer treatment failure.

## 4. CAAs in Breast Cancer Therapy

### 4.1. Therapeutic Resistance

**Figure 4:**
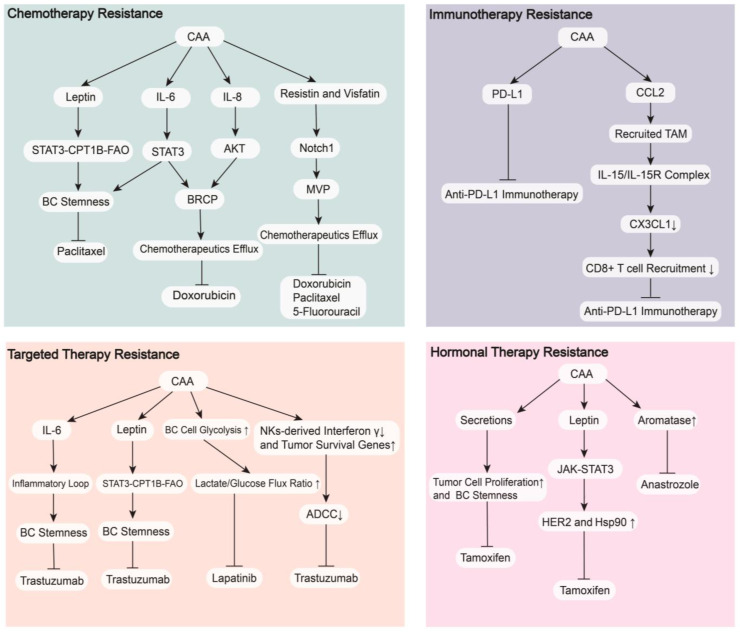
CAAs lead to therapeutic resistance in BC. CAAs secrete IL-6, IL-8, leptin, resistin and visfatin to cause chemotherapy resistance in BC. CAAs secrete PD-L1 and CCL2 to cause immunotherapy resistance in BC. CAAs secreting IL-6 and leptin, increasing BC cell glycolysis, decreasing NK-derived interferon, and increasing tumor survival genes cause targeted therapy resistance in BC. CAAs secreting leptin and other secretions and increasing expression of aromatase cause hormonal therapy resistance in BC. Abbreviations: BC, breast cancer; CAAs, cancer-associated adipocytes; IL-6, interleukin-6; IL-8, interleukin-8; HER2, human epidermal growth factor receptor 2; ADCC, antibody-dependent cellular cytotoxicity; BRCP, breast cancer resistance protein; Hsp90, heat-shock protein 90; STAT, signal transducer and activator of transcription 3; CPT1, carnitine palmitoyl transferase 1; FAO, fatty acid oxidation; AKT, protein kinase B; MVP, major vault protein; JAK, Janus tyrosine kinase; NKs, natural killer cells; PD-L1, programmed death-ligand 1; IL-15, interleukin-15; IL-15R, interleukin-15 receptor; CCL2, C-C motif chemokine ligand 2; CX3CL1, C-X3-C motif chemokine ligand 1; CD8+, cluster of differentiation 8 positive.

Although there are increasing numbers of agents that contribute greatly to the treatment of BC in addition to surgery, therapeutic resistance greatly harms the therapeutic effectiveness, shortens the survival time, and weakens the quality of life of BC patients. The interaction between CAAs and BC cells is also involved in resistance to multiple treatments, including chemotherapy, targeted therapy, hormonal therapy, and immune therapy (Figure 4). Known mechanisms primarily include activation of drug transport proteins, escape of treatment-induced apoptosis, enhancement of DNA repair, alteration of drug metabolism, and changes in the TME [142]. Therefore, we may find new ways to deal with therapeutic resistance in BC by performing profound research on CAAs.

#### 4.1.1. Chemotherapy Resistance

CAAs increase the release of leptin to promote the stemness of BC cells by activating the STAT3-CPT1B-FAO axis, which enhances the resistance to paclitaxel. Blockade of FAO and/or leptin makes BC cells sensitive to chemotherapy [143]. Major vault protein (MVP) is a transport-associated protein that is upregulated by adipocyte-derived resistin and visfatin via activation of the Notch1 signaling pathway in BC [79,144]. After co-cultured with human mammary adipocytes, high MVP expression in BC led to resistance to doxorubicin (with cross-resistance to paclitaxel and 5-fluorouracil) by accelerating chemotherapeutic efflux from BC cells [145]. Increased secretion of IL-8 and IL-6 promotes the expression of BC resistance protein (BRCP) by activating the AKT and STAT3 signaling pathways, respectively, which induce doxorubicin treatment resistance by accelerating efflux from BC cells. IL-6 activates the STAT3 signaling to increase BC stemness, which results in resistance to paclitaxel [146].

#### 4.1.2. Targeted Therapy Resistance

CAAs increase the release of IL-6 to expand the cancer stem cell population by activating an IL-6 inflammatory loop, which results in anti-trastuzumab effects in HER2+ BC patients that is overcome by an IL-6 receptor antibody [147]. CAAs enhance the secretion of leptin, which induces BC stemness and causes trastuzumab resistance by activating the STAT3-CPT1B-FAO axis [148]. CAAs decrease the trastuzumab-mediated antibody-dependent cellular cytotoxicity (ADCC) effect in HER2+ BC patients by decreasing the interferon γ secreted by NKs and modifying the tumor cell phenotype via the upregulation of some survival genes, such as DUSP5, MCL1 and CEBPD [149]. CAAs also increase glycolysis in BC cell metabolism, which results in higher lactate/glucose flux ratio and leads to resistance to lapatinib in HER2+ BC patients [150].

#### 4.1.3. Hormonal Therapy Resistance

When co-cultured with MCF7 cells, CAAs increased the levels of CYP19A1 mRNA to upregulate the expression of aromatase, which reduced their sensitivity to the aromatase inhibitor anastrozole and promoted the growth and progression of BC [151]. CAAs enhance the release of leptin to increase the expression of HER2 and heat-shock protein 90 (Hsp90) by activating the JAK/STAT3 signaling pathways, which reduces sensitivity to tamoxifen in ER+ BC. The Hsp90 inhibitor 17-AAG completely abrogated this process [152]. A recent study demonstrated that CAAs and their secretions suppressed the antiproliferative effect of tamoxifen by upregulating BC cells stemness and increasing BC cell proliferation [153].

#### 4.1.4. Immunotherapy Resistance

PD-L1 expression in CAAs prevents anti-PD-L1 antibodies from activating important antitumor functions of CD8+ T lymphocytes, which results in BC resistance to anti-PD-L1 immunotherapy [141]. CAAs increase the levels of CCL2 to recruit TAMs, which release the IL-15/IL-15 receptor complex to reduce C-X3-C motif chemokine ligand 1 (CX3CL1) expression in tumor cells. This reduced expression inhibits the recruitment of CD8+ T cells, which increases resistance to anti-PD-1 immunotherapy in BC and promotes BC progression. Administration of an IL-15/IL-15 receptor complex blocking peptide markedly suppressed this process [154].

Overall, CAAs secrete multiple adipokines and cytokines to activate related signaling pathways in tumor cells or model the pathological status of the TME, which are responsible for BC therapeutic resistance. However, the mechanisms governing the drug resistance between CAAs and BC are not fully understood, and more fundamental and clinical research must be performed.

### 4.2. Targeting CAAs Strategies

The interaction between CAAs and BC promotes tumor progression and induces treatment resistance, which indicates that targeting CAAs themselves or the secretome in CAAs may be a new strategy to further improve BC treatment effects [155]. These drugs are divided into different categories: drugs targeting the formation of CAAs, drugs targeting immune and metabolism changes in CAAs, and drugs targeting cytokines and adipokines secreted by CAAs.

#### 4.2.1. Drugs Targeting the Formation of CAAs

Metformin inhibits ADSC differentiation into adipocytes, which inhibits BC proliferation and invasion [156]. Renin (captopril)-angiotensin (telmisartan) inhibitors inhibits ADSC differentiation into adipocytes in vitro to decrease the invasiveness of MDA-MB-231 BC cell lines [157]. Green tea-derived epigallocatechin-3-gallate (EGCG) inhibits ADSC differentiation into adipocytes, which prevents the invasive phenotype of TNBC [158].

#### 4.2.2. Drugs Targeting Immune and Metabolism Changes in CAAs

The PPARγ antagonist GW9662 inhibited the progression and metastasis of BC by selectively reducing PD-L1 expression in mouse adipose tissue and enhanced the antitumor effect of CD8+ T cells in homozygous BC models [141]. Aspirin modifies the metabolomics and fatty acid composition of 3T3-L1 adipocytes by suppressing lipogenesis and oxidative stress to inhibit obesity-associated inflammation, which ultimately inhibits BC cell growth and migration [159]. The neutralizing antibodies JC63.1 and FA6.152 suppress the expression of the fatty acid transporter CD36 in CAAs around BC cells, which inhibits the metastasis of BC [160].

#### 4.2.3. Drugs Targeting Cytokines and Adipokines Secreted by CAAs

Some drugs focus on specific cytokines or adipokines, but other drugs reduce the secretion of multiple factors from CAAs. The following section discusses some drugs focusing on leptin, adiponectin, IL-6, CCL2, CCL5, IL-1β, and G-CSF. The adiponectin receptor agonist ADP355 inhibits BC cell proliferation by affecting various signaling pathways, such as ERK1/2 and STAT3, in vitro and in vivo [161]. Treating BC cells with the PPARγ agonist rosiglitazone increases adiponectin expression in adipocytes, which inhibits the migration and invasion of BC [162]. Pegylated leptin peptide receptor antagonist 2 (PEG-LPrA2) reduces the expression of leptin receptor and VEGFR2 in tumor cells, which inhibits the proliferation, angiogenesis and metastasis of ER+ and ER- BC [163]. Repurposing tocilizumab (IL-6R monoclonal antibody) may be a strategy to treat TNBC that overexpress IL-6R by reducing the chromosomal instability of tumor cells [164]. TAp73 (one kind of tumor suppressor) inhibits NF-κB-mediated CCL2 secretion and TAM recruitment, which reduces BC growth and metastasis [165]. A designed protein trap that binds CCL2 with high affinity and specificity reduces CCL2 levels in the TME, which decreases the population of immunosuppressive M2 macrophages and MDSCs and inhibits the metastasis of TNBC [166]. The recruitment and polarization of TAMs by the CCL5 axis is blocked by maraviroc (FDA-approved CCR5 inhibitor), which inhibits BC growth and metastasis and improves the overall survival rate for breast malignant phyllodes tumor [167]. Anakinra or canakinumab (anti-IL-1β monoclonal antibody) reduces EMT of BC cells and the number of tumor cells moving into the circulation, which ultimately reduces the bone metastasis of BC [168]. Metformin neutralizes G-CSF secreted by adipocytes in the breast cancer TME, which reduces immunosuppression, intratumor vascularization, angiogenesis, and progression of BC [169].

Drugs that simultaneously reduce the secretion of multiple cytokines and adipokines from CAAs may be more helpful in BC treatment. Eicosatetraenoic acid (EPA) is an anti-inflammatory n-3 polyunsaturated fatty acid, which reduces CAA-related inflammatory factors, such as IL-6, leptin, and PGE2, and reduces inflammation, glycolysis, and the motility of BC cells. It suggests that EPA may be an underlying therapeutic agent for BC [170]. According to a recent review, thymoquinone protected against the inflammatory TME by reducing the pro-inflammatory factors produced by CAAs such as IL-6 and IL-1β, which prevented the progression of TNBC [171]. EGCG from a polyphenol-rich diet inhibited the CAA-like phenotype of ADSCs when these cells were co-cultured with TNBC and reduced the secretion of CCL2, CCL5, IL-1β, IL-6, and VEGF, which reduced the invasion and metastasis of BC [172]. In general, Strategies targeting the secretome in CAAs attenuate the therapeutic resistance, reduce inflammation in the TME, change the immunosuppressive status of TIME, and inhibit the progression and metastasis of BC. Further research on the secretome in CAAs may identify more strategies to help BC patients in the future.

## 5. Conclusions

Breast cancer cells secrete WNT proteins and exosomes to inhibit the expression of PPARγ in adjacent adipocytes to form CAAs, and CAAs obtain a malignant phenotype in the TME compared with normal adipocytes. CAAs increase the secretion of cytokines and adipokines, such as leptin, IL-6, IL-1β, CCL2, and CCL5, and decrease the secretion of adiponectin, which regulate signaling pathways in BC cells and interact with other cells in the TME. CAAs remodel the BC ECM, increase aromatase expression in BC, cause various BC metabolic changes, and induce an immunosuppressive TME, which ultimately promote tumorigenesis, progression, and treatment resistance in BC. Targeting CAAs formation, CAAs themselves, and their secretome provide a promising strategy to improve BC treatment outcomes.

## References

[1] Sung H., Ferlay J., Siegel R.L., Laversanne M., Soerjomataram I., Jemal A., Bray F. (2021). Global Cancer Statistics 2020: GLOBOCAN Estimates of Incidence and Mortality Worldwide for 36 Cancers in 185 Countries. CA Cancer. J. Clin..

[2] Danenberg E., Bardwell H., Zanotelli V.R.T., Provenzano E., Chin S., Rueda O.M., Green A., Rakha E., Aparicio S., Ellis I.O. (2022). Breast tumor microenvironment structures are associated with genomic features and clinical outcome. Nat. Genet..

[3] Singleton D.C., Macann A., Wilson W.R. (2021). Therapeutic targeting of the hypoxic tumour microenvironment. Nat. Rev. Clin. Oncol..

[4] Zhang Q., Wang W., Zhou Q., Chen C., Yuan W., Liu J., Li X., Sun Z. (2020). Roles of circRNAs in the tumour microenvironment. Mol. Cancer.

[5] Zhou H., Wang M., Zhang Y., Su Q., Xie Z., Chen X., Yan R., Li P., Li T., Qin X. (2022). Functions and clinical significance of mechanical tumor microenvironment: Cancer cell sensing, mechanobiology and metastasis. Cancer Commun..

[6] Bejarano L., Jordāo M.J.C., Joyce J.A. (2021). Therapeutic Targeting of the Tumor Microenvironment. Cancer Discov..

[7] Wu Q., Li B., Li Z., Li J., Sun S., Sun S. (2019). Cancer-associated adipocytes: Key players in breast cancer progression. J. Hematol. Oncol..

[8] Fraser J.K., Wulur I., Alfonso Z., Hedrick M.H. (2006). Fat tissue: An underappreciated source of stem cells for biotechnology. Trends Biotechnol..

[9] Gregoire F.M., Smas C.M., Sul H.S. (1998). Understanding Adipocyte Differentiation. Physiol. Rev..

[10] Zhao C., Wu M., Zeng N., Xiong M., Hu W., Lv W., Yi Y., Zhang Q., Wu Y. (2020). Cancer-associated adipocytes: Emerging supporters in breast cancer. J. Exp. Clin. Cancer Res..

[11] Rybinska I., Mangano N., Tagliabue E., Triulzi T. (2021). Cancer-Associated Adipocytes in Breast Cancer: Causes and Consequences. Int. J. Mol. Sci..

[12] Protani M., Coory M., Martin J.H. (2010). Effect of obesity on survival of women with breast cancer: Systematic review and meta-analysis. Breast Cancer Res. Treat..

[13] Namazi N., Irandoost P., Heshmati J., Larijani B., Azadbakht L. (2019). The association between fat mass and the risk of breast cancer: A systematic review and meta-analysis. Clin. Nutr..

[14] Rybinska I., Agresti R., Trapani A., Tagliabue E., Triulzi T. (2020). Adipocytes in Breast Cancer, the Thick and the Thin. Cells.

[15] Dias A.S., Almeida C.R., Helguero L.A., Duarte I.F. (2019). Metabolic crosstalk in the breast cancer microenvironment. Eur. J. Cancer.

[16] Tang Q.Q., Lane M.D. (2012). Adipogenesis: From stem cell to adipocyte. Annu. Rev. Biochem..

[17] Hall J.A., Ramachandran D., Roh H.C., DiSpirito J.R., Belchior T., Zushin P.H., Palmer C., Hong S., Mina A.I., Liu B. (2020). Obesity-Linked PPARγ S273 Phosphorylation Promotes Insulin Resistance through Growth Differentiation Factor 3. Cell Metab..

[18] Madsen M.S., Siersbæk R., Boergesen M., Nielsen R., Mandrup S. (2014). Peroxisome Proliferator-Activated Receptor γ and C/EBPα Synergistically Activate Key Metabolic Adipocyte Genes by Assisted Loading. Mol. Cell. Biol..

[19] Xu X., Zhang M., Xu F., Jiang S. (2020). Wnt signaling in breast cancer: Biological mechanisms, challenges and opportunities. Mol. Cancer.

[20] Ross S.E., Hemati N., Longo K.A., Bennett C.N., Lucas P.C., Erickson R.L., Macdougald O.A. (2000). Inhibition of Adipogenesis by Wnt Signaling. Science.

[21] Gustafson B., Smith U. (2010). Activation of Canonical Wingless-type MMTV Integration Site Family (Wnt) Signaling in Mature Adipocytes Increases β-Catenin Levels and Leads to Cell Dedifferentiation and Insulin Resistance. J. Biol. Chem..

[22] Zoico E., Darra E., Rizzatti V., Budui S., Franceschetti G., Mazzali G., Rossi A.P., Fantin F., Menegazzi M., Cinti S. (2016). Adipocytes WNT5a mediated dedifferentiation: A possible target in pancreatic cancer microenvironment. Oncotarget.

[23] Topol L., Jiang X., Choi H., Garrett-Beal L., Carolan P.J., Yang Y. (2003). Wnt-5a inhibits the canonical Wnt pathway by promoting GSK-3–independent β-catenin degradation. J. Cell Biol..

[24] Wu Q., Li J., Li Z., Sun S., Zhu S., Wang L., Wu J., Yuan J., Zhang Y., Sun S. (2019). Exosomes from the tumour-adipocyte interplay stimulate beige/brown differentiation and reprogram metabolism in stromal adipocytes to promote tumour progression. J. Exp. Clin. Cancer Res..

[25] Wu Q., Sun S., Li Z., Yang Q., Li B., Zhu S., Wang L., Wu J., Yuan J., Yang C. (2018). Tumour-originated exosomal miR-155 triggers cancer-associated cachexia to promote tumour progression. Mol. Cancer.

[26] Wang S., Su X., Xu M., Xiao X., Li X., Li H., Keating A., Zhao R.C. (2019). Exosomes secreted by mesenchymal stromal/stem cell-derived adipocytes promote breast cancer cell growth via activation of Hippo signaling pathway. Stem Cell Res. Ther..

[27] Zhou X., Zhang J., Lv W., Zhao C., Xia Y., Wu Y., Zhang Q. (2022). The pleiotropic roles of adipocyte secretome in remodeling breast cancer. J. Exp. Clin. Cancer Res..

[28] Mubtasim N., Moustaid-Moussa N., Gollahon L. (2022). The Complex Biology of the Obesity-Induced, Metastasis-Promoting Tumor Microenvironment in Breast Cancer. Int. J. Mol. Sci..

[29] Lyu X., Zhang Q., Fares H.M., Wang Y., Han Y., Sun L. (2022). Contribution of adipocytes in the tumor microenvironment to breast cancer metabolism. Cancer Lett..

[30] Zahid H., Simpson E.R., Brown K.A. (2016). Inflammation, dysregulated metabolism and aromatase in obesity and breast cancer. Curr. Opin. Pharm..

[31] García-Estevez L., González-Martínez S., Moreno-Bueno G. (2021). The Leptin Axis and Its Association with the Adaptive Immune System in Breast Cancer. Front. Immunol..

[32] Sánchez-Jiménez F., Pérez-Pérez A., de la Cruz-Merino L., Sánchez-Margalet V. (2019). Obesity and Breast Cancer: Role of Leptin. Front. Oncol..

[33] Catalano S., Giordano C., Rizza P., Gu G., Barone I., Bonofiglio D., Giordano F., Malivindi R., Gaccione D., Lanzino M. (2009). Evidence that leptin through STAT and CREB signaling enhances cyclin D1 expression and promotes human endometrial cancer proliferation. J. Cell Physiol..

[34] Chen P., Wang B., Li M., Cui C., Liu F., Gao Y. (2022). Celastrol inhibits the proliferation and migration of MCF-7 cells through the leptin-triggered PI3K/AKT pathway. Comput. Struct. Biotechnol. J..

[35] Zhang C., Yue C., Herrmann A., Song J., Egelston C., Wang T., Zhang Z., Li W., Lee H., Aftabizadeh M. (2020). STAT3 Activation-Induced Fatty Acid Oxidation in CD8+ T Effector Cells Is Critical for Obesity-Promoted Breast Tumor Growth. Cell Metab..

[36] Li S., Wei X., Zhan X., He J., Zeng Y., Tian X., Yuan S., Sun L. (2020). Adipocyte-Derived Leptin Promotes PAI-1-Mediated Breast Cancer Metastasis in a STAT3/miR-34a Dependent Manner. Cancers.

[37] Gonzalez R.R., Cherfils S., Escobar M., Yoo J.H., Carino C., Styer A.K., Sullivan B.T., Sakamoto H., Olawaiye A., Serikawa T. (2006). Leptin Signaling Promotes the Growth of Mammary Tumors and Increases the Expression of Vascular Endothelial Growth Factor (VEGF) and Its Receptor Type Two (VEGF-R2). J. Biol. Chem..

[38] Kim H.G., Jin S.W., Kim Y.A., Khanal T., Lee G.H., Kim S.J., Rhee S.D., Chung Y.C., Hwang Y.J., Jeong T.C. (2017). Leptin induces CREB-dependent aromatase activation through COX-2 expression in breast cancer cells. Food Chem. Toxicol..

[39] Luo Y., Li H., Zhang Y., Wu Y., Shen D., Che Y. (2021). Combination of Endogenous Estradiol and Adipokine Leptin in Breast Cancer Risk and Prognosis Assessment in Postmenopausal Chinese Women. Front. Endocrinol..

[40] Van Gemert W.A., May A.M., Schuit A.J., Oosterhof B.Y.M., Peeters P.H., Monninkhof E.M. (2016). Effect of Weight Loss with or without Exercise on Inflammatory Markers and Adipokines in Postmenopausal Women: The SHAPE-2 Trial, A Randomized Controlled Trial. Cancer Epidemiol. Biomark. Prev..

[41] Iyengar N.M., Arthur R., Manson J.E., Chlebowski R.T., Kroenke C.H., Peterson L., Cheng T.D., Feliciano E.C., Lane D., Luo J. (2019). Association of Body Fat and Risk of Breast Cancer in Postmenopausal Women with Normal Body Mass Index. JAMA Oncol..

[42] Sturgeon K., Digiovanni L., Good J., Salvatore D., Fenderson D., Domchek S., Stopfer J., Galantino M.L., Bryan C., Hwang W. (2016). Exercise-Induced Dose-Response Alterations in Adiponectin and Leptin Levels Are Dependent on Body Fat Changes in Women at Risk for Breast Cancer. Cancer. Epidemiol. Biomark. Prev..

[43] Choi J., Cha Y.J., Koo J.S. (2018). Adipocyte biology in breast cancer: From silent bystander to active facilitator. Prog. Lipid Res..

[44] Shahril M.R., Zakarai N.S., Appannah G., Nurnazahiah A., Mohamed H.J.J., Ahmad A., Lua P.L., Fenech M. (2021). ‘Energy-Dense, High-SFA and Low-Fiber’ Dietary Pattern Lowered Adiponectin but Not Leptin Concentration of Breast Cancer Survivors. Nutrients.

[45] Andò S., Naimo G.D., Gelsomino L., Catalano S., Mauro L. (2020). Novel insights into adiponectin action in breast cancer: Evidence of its mechanistic effects mediated by ERα expression. Obes. Rev..

[46] Pham D.V., Park P.H. (2022). Adiponectin triggers breast cancer cell death via fatty acid metabolic reprogramming. J. Exp. Clin. Cancer Res..

[47] Chung S.J., Nagaraju G.P., Nagalingam A., Muniraj N., Kuppusamy P., Walker A., Woo J., Gyorffy B., Gabrielson E., Saxena N.K. (2017). ADIPOQ/adiponectin induces cytotoxic autophagy in breast cancer cells through STK11/LKB1-mediated activation of the AMPK-ULK1 axis. Autophagy.

[48] Macis D., Guerrieri-Gonzaga A., Gandini S. (2014). Circulating adiponectin and breast cancer risk: A systematic review and meta-analysis. Int. J. Epidemiol..

[49] Yoon Y.S., Kwon A.R., Lee Y.K., Oh S.W. (2019). Circulating adipokines and risk of obesity related cancers: A systematic review and meta-analysis. Obes. Res. Clin. Pract..

[50] Hirano T. (2021). IL-6 in inflammation, autoimmunity and cancer. Int. Immunol..

[51] Kim H.S., Jung M., Choi S.K., Woo J., Piao Y.J., Hwang E.H., Kim H., Kim S.J., Moon W.K. (2018). IL-6-mediated cross-talk between human preadipocytes and ductal carcinoma in situ in breast cancer progression. J. Exp. Clin. Cancer Res..

[52] He J., Wei X., Li S., Liu Y., Hu H., Li Z., Kuang X., Wang L., Shi X., Yuan S. (2018). Adipocyte-derived IL-6 and leptin promote breast Cancer metastasis via upregulation of Lysyl Hydroxylase-2 expression. Cell Commun. Signal..

[53] Weng Y., Tseng H., Chen Y., Shen P., Al Haq A.T., Chen L., Tung Y., Hsu H. (2019). MCT-1/miR-34a/IL-6/IL-6R signaling axis promotes EMT progression, cancer stemness and M2 macrophage polarization in triple-negative breast cancer. Mol. Cancer.

[54] Siersbæk R., Scabia V., Nagarajan S., Chernukhin I., Papachristou E.K., Broome R., Johnston S.J., Joosten S.E.P., Green A.R., Kumar S. (2020). IL6/STAT3 Signaling Hijacks Estrogen Receptor α Enhancers to Drive Breast Cancer Metastasis. Cancer Cell.

[55] Benoy I., Salgado R., Colpaert C., Weytjens R., Vermeulen P.B., Dirix L.Y. (2002). Serum Interleukin 6, Plasma VEGF, Serum VEGF, and VEGF Platelet Load in Breast Cancer Patients. Clin. Breast Cancer.

[56] Salgado R., Junius S., Benoy I., Van Dam P., Vermeulen P., Van Marck E., Huget P., Dirix L.Y. (2003). Circulating interleukin-6 predicts survival in patients with metastatic breast cancer. Int. J. Cancer.

[57] Gupta N., Goswami B., Mittal P. (2012). Effect of standard anthracycline based neoadjuvant chemotherapy on circulating levels of serum IL-6 in patients of locally advanced carcinoma breast—A prospective study. Int. J. Surg..

[58] Midavaine É., Côté J., Sarret P. (2021). The multifaceted roles of the chemokines CCL2 and CXCL12 in osteophilic metastatic cancers. Cancer Metast. Rev..

[59] Arendt L.M., McCready J., Keller P.J., Baker D.D., Naber S.P., Seewaldt V., Kuperwasser C. (2013). Obesity Promotes Breast Cancer by CCL2-Mediated Macrophage Recruitment and Angiogenesis. Cancer Res..

[60] Bonapace L., Coissieux M., Wyckoff J., Mertz K.D., Varga Z., Junt T., Bentires-Alj M. (2014). Cessation of CCL2 inhibition accelerates breast cancer metastasis by promoting angiogenesis. Nature.

[61] Müller A.K., Köhler U.A., Trzebanski S., Vinik Y., Raj H.M., Girault J.A., Ben Chetrit N., Maraver A., Jung S., Lev S. (2022). Mouse Modeling Dissecting Macrophage–Breast Cancer Communication Uncovered Roles of PYK2 in Macrophage Recruitment and Breast Tumorigenesis. Adv. Sci..

[62] Cao Y., Cao W., Qiu Y., Zhou Y., Guo Q., Gao Y., Lu N. (2020). Oroxylin A suppresses ACTN1 expression to inactivate cancer-associated fibroblasts and restrain breast cancer metastasis. Pharm. Res..

[63] Rogic A., Pant I., Grumolato L., Fernandez-Rodriguez R., Edwards A., Das S., Sun A., Yao S., Qiao R., Jaffer S. (2021). High endogenous CCL2 expression promotes the aggressive phenotype of human inflammatory breast cancer. Nat. Commun..

[64] Velasco-Velázquez M., Xolalpa W., Pestell R.G. (2014). The potential to target CCL5/CCR5 in breast cancer. Expert. Opin. Ther. Targets.

[65] Karnoub A.E., Dash A.B., Vo A.P., Sullivan A., Brooks M.W., Bell G.W., Richardson A.L., Polyak K., Tubo R., Weinberg R.A. (2007). Mesenchymal stem cells within tumour stroma promote breast cancer metastasis. Nature.

[66] Song X., Zhou X., Qin Y., Yang J., Wang Y., Sun Z., Yu K., Zhang S., Liu S. (2018). Emodin inhibits epithelial-mesenchymal transition and metastasis of triple negative breast cancer via antagonism of CC-chemokine ligand 5 secreted from adipocytes. Int. J. Mol. Med..

[67] An G., Wu F., Huang S., Feng L., Bai J., Gu S., Zhao X. (2019). Effects of CCL5 on the biological behavior of breast cancer and the mechanisms of its interaction with tumor-associated macrophages. Oncol. Rep..

[68] Ban Y., Mai J., Li X., Mitchell-Flack M., Zhang T., Zhang L., Chouchane L., Ferrari M., Shen H., Ma X. (2017). Targeting Autocrine CCL5–CCR5 Axis Reprograms Immunosuppressive Myeloid Cells and Reinvigorates Antitumor Immunity. Cancer Res..

[69] Zhang Q., Qin J., Zhong L., Gong L., Zhang B., Zhang Y., Gao W. (2015). CCL5-Mediated Th2 Immune Polarization Promotes Metastasis in Luminal Breast Cancer. Cancer Res..

[70] Sax M.J., Gasch C., Athota V.R., Freeman R., Rasighaemi P., Westcott D.E., Day C.J., Nikolic I., Elsworth B., Wei M. (2016). Cancer cell CCL5 mediates bone marrow independent angiogenesis in breast cancer. Oncotarget.

[71] Zazo S., González-Alonso P., Martín-Aparicio E., Chamizo C., Luque M., Sanz-Álvarez M., Mínguez P., Gómez-López G., Cristóbal I., Caramés C. (2020). Autocrine CCL5 Effect Mediates Trastuzumab Resistance by ERK Pathway Activation in HER2-Positive Breast Cancer. Mol. Cancer Ther..

[72] Zhou J., Tulotta C., Ottewell P.D. (2022). IL-1β in breast cancer bone metastasis. J. Expert. Rev. Mol. Med..

[73] Perrier S., Caldefie-Chézet F., Vasson M. (2009). IL-1 family in breast cancer: Potential interplay with leptin and other adipocytokines. FEBS Lett..

[74] Pein M., Insua-Rodríguez J., Hongu T., Riedel A., Meier J., Wiedmann L., Decker K., Essers M.A.G., Sinn H., Spaich S. (2020). Metastasis-initiating cells induce and exploit a fibroblast niche to fuel malignant colonization of the lungs. Nat. Commun..

[75] Pradhan A.K., Maji S., Bhoopathi P., Talukdar S., Mannangatti P., Guo C., Wang X., Cartagena L.C., Idowu M., Landry J.W. (2021). Pharmacological inhibition of MDA-9/Syntenin blocks breast cancer metastasis through suppression of IL-1β. Proc. Natl. Acad. Sci. USA.

[76] Kolb R., Kluz P., Tan Z.W., Borcherding N., Bormann N., Vishwakarma A., Balcziak L., Zhu P., Davies B.S., Gourronc F. (2019). Obesity-associated inflammation promotes angiogenesis and breast cancer via angiopoietin-like 4. Oncogene.

[77] Dias J.A., Fredrikson G.N., Ericson U., Gullberg B., Hedblad B., Engström G., Borgquist S., Nilsson J., Wirfält E. (2016). Low-Grade Inflammation, Oxidative Stress and Risk of Invasive Post-Menopausal Breast Cancer—A Nested Case-Control Study from the Malmö Diet and Cancer Cohort. PLoS ONE.

[78] Vilsmaier T., Rack B., König A., Friese K., Janni W., Jeschke U., Acher T.W. (2016). Influence of Circulating Tumour Cells on Production of IL-1α, IL-1β and IL-12 in Sera of Patients with Primary Diagnosis of Breast Cancer Before Treatment. Anticancer Res..

[79] Wang Y., Hung A.C., Lo S., Yuan S.F. (2021). Adipocytokines visfatin and resistin in breast cancer: Clinical relevance, biological mechanisms, and therapeutic potential. Cancer Lett..

[80] Gao Y., Chen X., He Q., Gimple R.C., Liao Y., Wang L., Wu R., Xie Q., Rich J.N., Shen K. (2020). Adipocytes promote breast tumorigenesis through TAZ-dependent secretion of Resistin. Proc. Natl. Acad. Sci. USA.

[81] Zhang X., Li M., Yin N., Zhang J. (2021). The Expression Regulation and Biological Function of Autotaxin. Cells.

[82] Zeng J., Hu J., Lian Y., Jiang Y., Chen B. (2018). SFRP5 is a target gene transcriptionally regulated by PPARgamma in 3T3-L1 adipocytes. Gene.

[83] Zhou W., Ye C., Li L., Liu L., Wang F., Yu L., Zhou F., Xiang Y., Wang Y., Yin G. (2020). Adipocyte-derived SFRP5 inhibits breast cancer cells migration and invasion through Wnt and epithelial-mesenchymal transition signaling pathways. Chin. J. Cancer Res..

[84] Wu Q., Li B., Li J., Sun S., Yuan J., Sun S. (2021). Cancer-associated adipocytes as immunomodulators in cancer. Biomark. Res..

[85] Queen M.M., Ryan R.E., Holzer R.G., Keller-Peck C.R., Jorcyk C.L. (2005). Breast Cancer Cells Stimulate Neutrophils to Produce Oncostatin M: Potential Implications for Tumor Progression. Cancer Res..

[86] Bell L.N., Cai L., Johnstone B.H., Traktuev D.O., March K.L., Considine R.V. (2008). A central role for hepatocyte growth factor in adipose tissue angiogenesis. Am. J. Physiol.-Endoc. Metab..

[87] Sam M.R., Elliott B.E., Mueller C.R. (2007). A novel activating role of SRC and STAT3 on HGF transcription in human breast cancer cells. Mol. Cancer.

[88] Yamashita J., Ogawa M., Yamashita S., Nomura K., Kuramoto M., Saishoji T., Shin S. (1994). Immunoreactive hepatocyte growth factor is a strong and independent predictor of recurrence and survival in human breast cancer. Cancer Res..

[89] Liu L., Wu Y., Zhang C., Zhou C., Li Y., Zeng Y., Zhang C., Li R., Luo D., Wang L. (2020). Cancer-associated adipocyte-derived G-CSF promotes breast cancer malignancy via Stat3 signaling. J. Mol. Cell. Biol..

[90] Su X., Xu Y., Fox G.C., Xiang J., Kwakwa K.A., Davis J.L., Belle J.I., Lee W., Wong W.H., Fontana F. (2021). Breast cancer–derived GM-CSF regulates arginase 1 in myeloid cells to promote an immunosuppressive microenvironment. J. Clin. Investig..

[91] Ferrara N., Gerber H.P., LeCouter J. (2003). The biology of VEGF and its receptors. Nat. Med..

[92] Foekens J.A., Peters H.A., Grebenchtchikov N., Look M.P., Meijer-van G.M., Geurts-Moespot A., van der Kwast T.H., Sweep C.G., Klijn J.G. (2001). High tumor levels of vascular endothelial growth factor predict poor response to systemic therapy in advanced breast cancer. Cancer Res..

[93] D’Esposito V., Passaretti F., Hammarstedt A., Liguoro D., Terracciano D., Molea G., Canta L., Miele C., Smith U., Beguinot F. (2012). Adipocyte-released insulin-like growth factor-1 is regulated by glucose and fatty acids and controls breast cancer cell growth in vitro. Diabetologia.

[94] Zeng L., Biernacka K.M., Holly J.M.P., Jarrett C., Morrison A.A., Morgan A., Winters Z.E., Foulstone E.J., Shield J.P., Perks C.M. (2010). Hyperglycaemia confers resistance to chemotherapy on breast cancer cells: The role of fatty acid synthase. Endocr.-Relat. Cancer.

[95] Zhou C., He X., Tong C., Li H., Xie C., Wu Y., Wang L., Yan X., Luo D., Tang Y. (2022). Cancer-associated adipocytes promote the invasion and metastasis in breast cancer through LIF/CXCLs positive feedback loop. Int. J. Biol. Sci..

[96] Cox T.R. (2021). The matrix in cancer. Nat. Rev. Cancer.

[97] Dinca S.C., Greiner D., Weidenfeld K., Bond L., Barkan D., Jorcyk C.L. (2021). Novel mechanism for OSM-promoted extracellular matrix remodeling in breast cancer: LOXL2 upregulation and subsequent ECM alignment. Breast Cancer Res..

[98] Juárez-Cruz J.C., Zuñiga-Eulogio M.D., Olea-Flores M., Castañeda-Saucedo E., Mendoza-Catalán M.Á., Ortuño-Pineda C., Moreno-Godínez M.E., Villegas-Comonfort S., Padilla-Benavides T., Navarro-Tito N. (2019). Leptin induces cell migration and invasion in a FAK-Src-dependent manner in breast cancer cells. Endocr. Connect..

[99] Iyengar P., Espina V., Williams T.W., Lin Y., Berry D., Jelicks L.A., Lee H., Temple K., Graves R., Pollard J. (2005). Adipocyte-derived collagen VI affects early mammary tumor progression in vivo, demonstrating a critical interaction in the tumor/stroma microenvironment. J. Clin. Investig..

[100] Vasiukov G., Novitskaya T., Zijlstra A., Owens P., Ye F., Zhao Z., Moses H.L., Blackwell T., Feoktistov I., Novitskiy S.V. (2020). Myeloid Cell–Derived TGFβ Signaling Regulates ECM Deposition in Mammary Carcinoma via Adenosine-Dependent Mechanisms. Cancer Res..

[101] Elia I., Rossi M., Stegen S., Broekaert D., Doglioni G., van Gorsel M., Boon R., Escalona-Noguero C., Torrekens S., Verfaillie C. (2019). Breast cancer cells rely on environmental pyruvate to shape the metastatic niche. Nature.

[102] Germain D. (2011). Estrogen Carcinogenesis in Breast Cancer. Endocrin. Metab. Clin..

[103] Van Landeghem A.A., Poortman J., Nabuurs M., Thijssen J.H. (1985). Endogenous concentration and subcellular distribution of androgens in normal and malignant human breast tissue. Cancer Res..

[104] Miller W.R. (1991). Aromatase activity in breast tissue. J. Steroid Biochem. Mol. Biol..

[105] Bhardwaj P., Au C.C., Benito-Martin A., Ladumor H., Oshchepkova S., Moges R., Brown K.A. (2019). Estrogens and breast cancer: Mechanisms involved in obesity-related development, growth and progression. J. Steroid Biochem. Mol. Biol..

[106] Wang X., Simpson E.R., Brown K.A. (2015). Aromatase overexpression in dysfunctional adipose tissue links obesity to postmenopausal breast cancer. J. Steroid Biochem. Mol. Biol..

[107] Ka N.L., Lim G.Y., Kim S.S., Hwang S., Han J., Lee Y.H., Lee M.O. (2022). Type I IFN stimulates IFI16-mediated aromatase expression in adipocytes that promotes E2-dependent growth of ER-positive breast cancer. Cell. Mol. Life Sci..

[108] Provance O.K., Lewis-Wambi J. (2019). Deciphering the role of interferon alpha signaling and microenvironment crosstalk in inflammatory breast cancer. Breast Cancer Res..

[109] Pouyssegur J., Marchiq I., Parks S.K., Durivault J., Zdralevic M., Vucetic M. (2022). ‘Warburg effect’ controls tumor growth, bacterial, viral infections and immunity—Genetic deconstruction and therapeutic perspectives. Semin. Cancer Biol..

[110] Brown K.A. (2021). Metabolic pathways in obesity-related breast cancer. Nat. Rev. Endocrinol..

[111] Hoxhaj G., Manning B.D. (2020). The PI3K-AKT network at the interface of oncogenic signalling and cancer metabolism. Nat. Rev. Cancer.

[112] Wang X., Docanto M.M., Sasano H., Lo C., Simpson E.R., Brown K.A. (2015). Prostaglandin E2 Inhibits p53 in Human Breast Adipose Stromal Cells: A Novel Mechanism for the Regulation of Aromatase in Obesity and Breast Cancer. Cancer Res..

[113] Frezza C. (2020). Metabolism and cancer: The future is now. Br. J. Cancer.

[114] Felmlee M.A., Jones R.S., Rodriguez-Cruz V., Follman K.E., Morris M.E. (2020). Monocarboxylate Transporters (SLC16): Function, Regulation, and Role in Health and Disease. Pharm. Rev..

[115] Sotgia F., Martinez-Outschoorn U.E., Howell A., Pestell R.G., Pavlides S., Lisanti M.P. (2012). Caveolin-1 and cancer metabolism in the tumor microenvironment: Markers, models, and mechanisms. Annu. Rev. Pathol..

[116] Sotgia F., Martinez-Outschoorn U.E., Pavlides S., Howell A., Pestell R.G., Lisanti M.P. (2011). Understanding the Warburg effect and the prognostic value of stromal caveolin-1 as a marker of a lethal tumor microenvironment. Breast Cancer Res..

[117] Singh R., Parveen M., Basgen J.M., Fazel S., Meshesha M.F., Thames E.C., Moore B., Martinez L., Howard C.B., Vergnes L. (2016). Increased Expression of Beige/Brown Adipose Markers from Host and Breast Cancer Cells Influence Xenograft Formation in Mice. Mol. Cancer Res..

[118] Grabner G.F., Xie H., Schweiger M., Zechner R. (2021). Lipolysis: Cellular mechanisms for lipid mobilization from fat stores. Nat. Metab..

[119] Currie E., Schulze A., Zechner R., Walther T.C., Farese R.V. (2013). Cellular Fatty Acid Metabolism and Cancer. Cell Metab..

[120] Glatz J.F., Luiken J.J. (2017). From fat to FAT (CD36/SR-B2): Understanding the regulation of cellular fatty acid uptake. Biochimie.

[121] Guaita-Esteruelas S., Saavedra-García P., Bosquet A., Borràs J., Girona J., Amiliano K., Rodríguez-Balada M., Heras M., Masana L., Gumà J. (2017). Adipose-Derived Fatty Acid-Binding Proteins Plasma Concentrations Are Increased in Breast Cancer Patients. Oncologist.

[122] Yang D., Li Y., Xing L., Tan Y., Sun J., Zeng B., Xiang T., Tan J., Ren G., Wang Y. (2018). Utilization of adipocyte-derived lipids and enhanced intracellular trafficking of fatty acids contribute to breast cancer progression. Cell Commun. Signal..

[123] Gyamfi J., Yeo J.H., Kwon D., Min B.S., Cha Y.J., Koo J.S., Jeong J., Lee J., Choi J. (2021). Interaction between CD36 and FABP4 modulates adipocyte-induced fatty acid import and metabolism in breast cancer. NPJ Breast Cancer.

[124] Wang Y.Y., Attané C., Milhas D., Dirat B., Dauvillier S., Guerard A., Gilhodes J., Lazar I., Alet N., Laurent V. (2017). Mammary adipocytes stimulate breast cancer invasion through metabolic remodeling of tumor cells. JCI Insight.

[125] Puig T., Aguilar H., Cufi S., Oliveras G., Turrado C., Ortega-Gutierrez S., Benhamu B., Lopez-Rodriguez M.L., Urruticoechea A., Colomer R. (2011). A novel inhibitor of fatty acid synthase shows activity against HER2+ breast cancer xenografts and is active in anti-HER2 drug-resistant cell lines. Breast Cancer Res..

[126] Simińska E., Koba M. (2016). Amino acid profiling as a method of discovering biomarkers for early diagnosis of cancer. Amino Acids.

[127] Munteanu R., Onaciu A., Moldovan C., Zimta A., Gulei D., Paradiso A., Lazar V., Berindan-Neagoe I. (2020). Adipocyte-Based Cell Therapy in Oncology: The Role of Cancer-Associated Adipocytes and Their Reinterpretation as Delivery Platforms. Pharmaceutics.

[128] Huang J., Diaz-Meco M.T., Moscat J. (2018). The macroenviromental control of cancer metabolism by p62. Cell Cycle.

[129] Balaban S., Shearer R.F., Lee L.S., van Geldermalsen M., Schreuder M., Shtein H.C., Cairns R., Thomas K.C., Fazakerley D.J., Grewal T. (2017). Adipocyte lipolysis links obesity to breast cancer growth: Adipocyte-derived fatty acids drive breast cancer cell proliferation and migration. Cancer Metab..

[130] Salimian Rizi B., Caneba C., Nowicka A., Nabiyar A.W., Liu X., Chen K., Klopp A., Nagrath D. (2015). Nitric Oxide Mediates Metabolic Coupling of Omentum-Derived Adipose Stroma to Ovarian and Endometrial Cancer Cells. Cancer Res..

[131] Pitt J.M., Marabelle A., Eggermont A., Soria J.C., Kroemer G., Zitvogel L. (2016). Targeting the tumor microenvironment: Removing obstruction to anticancer immune responses and immunotherapy. Ann. Oncol..

[132] Williams C.B., Yeh E.S., Soloff A.C. (2016). Tumor-associated macrophages: Unwitting accomplices in breast cancer malignancy. NPJ Breast Cancer.

[133] DeNardo D.G., Ruffell B. (2019). Macrophages as regulators of tumour immunity and immunotherapy. Nat. Rev. Immunol..

[134] Maller O., Drain A.P., Barrett A.S., Borgquist S., Ruffell B., Zakharevich I., Pham T.T., Gruosso T., Kuasne H., Lakins J.N. (2021). Tumour-associated macrophages drive stromal cell-dependent collagen crosslinking and stiffening to promote breast cancer aggression. Nat. Mater..

[135] Sossey-Alaoui K., Pluskota E., Bialkowska K., Szpak D., Parker Y., Morrison C.D., Lindner D.J., Schiemann W.P., Plow E.F. (2017). Kindlin-2 Regulates the Growth of Breast Cancer Tumors by Activating CSF-1–Mediated Macrophage Infiltration. Cancer Res..

[136] Corrêa L.H., Corrêa R., Farinasso C.M., de Sant Ana Dourado L.P., Magalhães K.G. (2017). Adipocytes and Macrophages Interplay in the Orchestration of Tumor Microenvironment: New Implications in Cancer Progression. Front. Immunol..

[137] Mu X., Shi W., Xu Y., Xu C., Zhao T., Geng B., Yang J., Pan J., Hu S., Zhang C. (2018). Tumor-derived lactate induces M2 macrophage polarization via the activation of the ERK/STAT3 signaling pathway in breast cancer. Cell Cycle.

[138] Engin A.B., Engin A., Gonul I.I. (2019). The effect of adipocyte–macrophage crosstalk in obesity-related breast cancer. J. Mol. Endocrinol..

[139] Vazquez Rodriguez G., Abrahamsson A., Jensen L.D.E., Dabrosin C. (2018). Adipocytes Promote Early Steps of Breast Cancer Cell Dissemination via Interleukin-8. Front. Immunol..

[140] Michelet X., Dyck L., Hogan A., Loftus R.M., Duquette D., Wei K., Beyaz S., Tavakkoli A., Foley C., Donnelly R. (2018). Metabolic reprogramming of natural killer cells in obesity limits antitumor responses. Nat. Immunol..

[141] Wu B., Sun X., Gupta H.B., Yuan B., Li J., Ge F., Chiang H.C., Zhang X., Zhang C., Zhang D. (2018). Adipose PD-L1 Modulates PD-1/PD-L1 Checkpoint Blockade Immunotherapy Efficacy in Breast Cancer. Oncoimmunology.

[142] Hillers-Ziemer L.E., Kuziel G., Williams A.E., Moore B.N., Arendt L.M. (2022). Breast cancer microenvironment and obesity: Challenges for therapy. Cancer. Metastasis Rev..

[143] Wang T., Fahrmann J.F., Lee H., Li Y., Tripathi S.C., Yue C., Zhang C., Lifshitz V., Song J., Yuan Y. (2018). JAK/STAT3-Regulated Fatty Acid β-Oxidation Is Critical for Breast Cancer Stem Cell Self-Renewal and Chemoresistance. Cell Metab..

[144] Xiao Y.S., Zeng D., Liang Y.K., Wu Y., Li M.F., Qi Y.Z., Wei X.L., Huang W.H., Chen M., Zhang G.J. (2019). Major vault protein is a direct target of Notch1 signaling and contributes to chemoresistance in triple-negative breast cancer cells. Cancer Lett..

[145] Lehuédé C., Li X., Dauvillier S., Vaysse C., Franchet C., Clement E., Esteve D., Longué M., Chaltiel L., Le Gonidec S. (2019). Adipocytes promote breast cancer resistance to chemotherapy, a process amplified by obesity: Role of the major vault protein (MVP). Breast Cancer Res..

[146] Plava J., Cihova M., Burikova M., Matuskova M., Kucerova L., Miklikova S. (2019). Recent advances in understanding tumor stroma-mediated chemoresistance in breast cancer. Mol. Cancer.

[147] Korkaya H., Kim G.I., Davis A., Malik F., Henry N.L., Ithimakin S., Quraishi A.A., Tawakkol N., D’Angelo R., Paulson A.K. (2012). Activation of an IL6 inflammatory loop mediates trastuzumab resistance in HER2+ breast cancer by expanding the cancer stem cell population. Mol. Cell.

[148] Han J., Qu H., Han M., Ding Y., Xie M., Hu J., Chen Y., Dong H. (2021). MSC-induced lncRNA AGAP2-AS1 promotes stemness and trastuzumab resistance through regulating CPT1 expression and fatty acid oxidation in breast cancer. Oncogene.

[149] Duong M.N., Cleret A., Matera E., Chettab K., Mathé D., Valsesia-Wittmann S., Clémenceau B., Dumontet C. (2015). Adipose cells promote resistance of breast cancer cells to trastuzumab-mediated antibody-dependent cellular cytotoxicity. Breast Cancer Res..

[150] Komurov K., Tseng J.T., Muller M., Seviour E.G., Moss T.J., Yang L., Nagrath D., Ram P.T. (2012). The glucose-deprivation network counteracts lapatinib-induced toxicity in resistant ErbB2-positive breast cancer cells. Mol. Syst. Biol..

[151] Morgan M.M., Arendt L.M., Alarid E.T., Beebe D.J., Johnson B.P. (2019). Mammary adipose stromal cells derived from obese women reduce sensitivity to the aromatase inhibitor anastrazole in an organotypic breast model. FASEB J..

[152] Giordano C., Vizza D., Panza S., Barone I., Bonofiglio D., Lanzino M., Sisci D., De Amicis F., Fuqua S.A.W., Catalano S. (2013). Leptin increases HER2 protein levels through a STAT3-mediated up-regulation of Hsp90 in breast cancer cells. Mol. Oncol..

[153] Delort L., Bougaret L., Cholet J., Vermerie M., Billard H., Decombat C., Bourgne C., Berger M., Dumontet C., Caldefie-Chezet F. (2019). Hormonal Therapy Resistance and Breast Cancer: Involvement of Adipocytes and Leptin. Nutrients..

[154] Zhang W., Zhang Q., Yang N., Shi Q., Su H., Lin T., He Z., Wang W., Guo H., Shen P. (2022). Crosstalk between IL-15Rα^+^ tumor-associated macrophages and breast cancer cells reduces CD8^+^ T cell recruitment. Cancer Commun..

[155] Cha Y.J., Koo J.S. (2018). Adipokines as therapeutic targets in breast cancer treatment. Expert Opin. Ther. Targets.

[156] Teufelsbauer M., Rath B., Plangger A., Staud C., Nanobashvili J., Huk I., Neumayer C., Hamilton G., Radtke C. (2020). Effects of metformin on adipose-derived stromal cell (ADSC) – Breast cancer cell lines interaction. Life Sci..

[157] Rasha F., Ramalingam L., Menikdiwela K., Hernandez A., Moussa H., Gollahon L., Layeequr Rahman R., Moustaid-Moussa N. (2020). Renin angiotensin system inhibition attenuates adipocyte-breast cancer cell interactions. Exp. Cell Res..

[158] Gonzalez S.N., Rodriguez T.S., Ouanouki A., El C.L., Annabi B. (2021). EGCG Inhibits Adipose-Derived Mesenchymal Stem Cells Differentiation into Adipocytes and Prevents a STAT3-Mediated Paracrine Oncogenic Control of Triple-Negative Breast Cancer Cell Invasive Phenotype. Molecules.

[159] Hsieh C., Chiu H., Wang C., Kuo C. (2020). Aspirin Modifies Inflammatory Mediators and Metabolomic Profiles and Contributes to the Suppression of Obesity-Associated Breast Cancer Cell Growth. Int. J. Mol. Sci..

[160] Pascual G., Avgustinova A., Mejetta S., Martín M., Castellanos A., Attolini C.S., Berenguer A., Prats N., Toll A., Hueto J.A. (2017). Targeting metastasis-initiating cells through the fatty acid receptor CD36. Nature.

[161] Otvos L.J., Haspinger E., La Russa F., Maspero F., Graziano P., Kovalszky I., Lovas S., Nama K., Hoffmann R., Knappe D. (2011). Design and development of a peptide-based adiponectin receptor agonist for cancer treatment. BMC Biotechnol..

[162] Taliaferro-Smith L., Nagalingam A., Knight B.B., Oberlick E., Saxena N.K., Sharma D. (2013). Integral Role of PTP1B in Adiponectin-Mediated Inhibition of Oncogenic Actions of Leptin in Breast Carcinogenesis. Neoplasia.

[163] Rene Gonzalez R., Watters A., Xu Y., Singh U.P., Mann D.R., Rueda B.R., Penichet M.L. (2009). Leptin-signaling inhibition results in efficient anti-tumor activity in estrogen receptor positive or negative breast cancer. Breast Cancer Res..

[164] Hong C., Schubert M., Tijhuis A.E., Requesens M., Roorda M., van den Brink A., Ruiz L.A., Bakker P.L., van der Sluis T., Pieters W. (2022). cGAS–STING drives the IL-6-dependent survival of chromosomally instable cancers. Nature.

[165] Wolfsberger J., Sakil H.A.M., Zhou L., van Bree N., Baldisseri E., de Souza Ferreira S., Zubillaga V., Stantic M., Fritz N., Hartman J. (2021). TAp73 represses NF-κB–mediated recruitment of tumor-associated macrophages in breast cancer. Proc. Natl. Acad. Sci. USA.

[166] Liu Y., Tiruthani K., Wang M., Zhou X., Qiu N., Xiong Y., Pecot C.V., Liu R., Huang L. (2021). Tumor-targeted gene therapy with lipid nanoparticles inhibits tumor-associated adipocytes and remodels the immunosuppressive tumor microenvironment in triple-negative breast cancer. Nanoscale Horiz..

[167] Nie Y., Huang H., Guo M., Chen J., Wu W., Li W., Xu X., Lin X., Fu W., Yao Y. (2019). Breast Phyllodes Tumors Recruit and Repolarize Tumor-Associated Macrophages via Secreting CCL5 to Promote Malignant Progression, Which Can Be Inhibited by CCR5 Inhibition Therapy. Clin. Cancer Res..

[168] Tulotta C., Lefley D.V., Freeman K., Gregory W.M., Hanby A.M., Heath P.R., Nutter F., Wilkinson J.M., Spicer-Hadlington A.R., Liu X. (2019). Endogenous Production of IL1B by Breast Cancer Cells Drives Metastasis and Colonization of the Bone Microenvironment. Clin. Cancer Res..

[169] Reggiani F., Labanca V., Mancuso P., Rabascio C., Talarico G., Orecchioni S., Manconi A., Bertolini F. (2017). Adipose Progenitor Cell Secretion of GM-CSF and MMP9 Promotes a Stromal and Immunological Microenvironment That Supports Breast Cancer Progression. Cancer Res..

[170] Al-Jawadi A., Rasha F., Ramalingam L., Alhaj S., Moussa H., Gollahon L., Dharmawardhane S., Moustaid-Moussa N. (2020). Protective effects of eicosapentaenoic acid in adipocyte-breast cancer cell cross talk. J. Nutr. Biochem..

[171] Adinew G.M., Taka E., Mochona B., Badisa R.B., Mazzio E.A., Elhag R., Soliman K.F.A. (2022). Therapeutic Potential of Thymoquinone in Triple-Negative Breast Cancer Prevention and Progression through the Modulation of the Tumor Microenvironment. Nutrients.

[172] Gonzalez Suarez N., Fernandez-Marrero Y., Torabidastgerdooei S., Annabi B. (2022). EGCG Prevents the Onset of an Inflammatory and Cancer-Associated Adipocyte-like Phenotype in Adipose-Derived Mesenchymal Stem/Stromal Cells in Response to the Triple-Negative Breast Cancer Secretome. Nutrients.

